# Safety of biological therapy in patients with rheumatoid arthritis in administrative health databases: A systematic review and meta-analysis

**DOI:** 10.3389/fphar.2022.928471

**Published:** 2022-08-11

**Authors:** Mariana Jorge de Queiroz, Caroline Tianeze de Castro, Flavia Caixeta Albuquerque, Celmário Castro Brandão, Leticia Farias Gerlack, Daniella Cristina Rodrigues Pereira, Sandra Castro Barros, Wenderson Walla Andrade, Ediane de Assis Bastos, Jessé de Nobrega Batista Azevedo, Roberto Carreiro, Mauricio Lima Barreto, Djanilson Barbosa Santos

**Affiliations:** ^1^ Department of Pharmaceutical Assistance and Strategic Inputs, Ministry of Health, Brasília, Brazil; ^2^Institute of Collective Health, Federal University of Bahia, Salvador, Brazil; ^3^ Fiocruz Brasília, Oswaldo Cruz Foundation (FIOCRUZ), Brasília, Brazil; ^4^ Center of Data and Knowledge Integration for Health (CIDACS), Gonçalo Moniz Institute, Oswaldo Cruz Foundation (FIOCRUZ), Salvador, Brazil; ^5^ Center for Health Sciences, Federal University of Recôncavo da Bahia, Santo Antônio de Jesus, Brazil

**Keywords:** rheumatoid arthritis, biological therapy, systematic review, meta-analysis, drug safety

## Abstract

**Background:** Rheumatoid arthritis (RA) is a systemic inflammatory disease that affects the synovial fluid of joints, tendons, and some extra-articular sites. Biologic agents have been highly effective and are comparable in reducing RA symptoms, slowing disease progression, and improving physical function; however, concerns have been raised about the risks of several potential adverse effects. Thus, this study aimed to assess the safety of biological therapy in patients with rheumatoid arthritis in observational studies using administrative health databases.

**Methods:** PubMed, Embase, Lilacs, Ovid, Scopus, and Web of Science were searched from inception to 21 October 2021. The analysis was divided into five groups: tumor necrosis factor inhibitors (TNFi) versus non-TNFi; TNFi versus csDMARDs; bDMARDs versus csDMARDs; abatacept versus bDMARDs; and TNFi versus Janus kinase inhibitors (JAKi). The adverse events were cancer, cardiovascular events, infection, herpes zoster, tuberculosis, and death. The methodological quality of the studies was assessed by the Newcastle-Ottawa Scale. A random-effects model estimated risk ratios with 95% confidence intervals.

**Results:** Thirty-one studies were eligible for inclusion in the present systematic review, published from 2014 to 2021. A total of 1,039,398 RA patients were assessed. The 31 studies evaluated eleven different biological drugs. No significant differences were found regarding safety between TNFi versus non-TNFi (RR 1.08; 95% CI 0.92–1.28; *p* < 0.01; I^2^ = 93.0%), TNFi versus csDMARDs (RR 0.91; 95% CI 0.75–1.10; *p* < 0.01; I^2^ = 87.0%), bDMARDs versus csDMARDs (RR 0.99; 95% CI 0.82–1.20; *p* < 0.01; I^2^ = 93.0%), abatacept versus bDMARDs (RR 0.80; 95% CI 0.54–1.18; *p* < 0.01; I^2^ = 90.0%), and TNFi versus JAKi (RR 3.54; 95% CI 0.30–42.09; *p* = 0.01; I^2^ = 81.0%). In the subgroup analysis, among studies comparing abatacept to TNFi, a lower risk of cardiovascular events was associated with abatacept (RR 0.37; 95% CI 0.24–0.55).

**Conclusion:** Our results do not suggest an increased risk of adverse events associated with biological therapy in treating RA patients, indicating a lower risk of cardiovascular events with abatacept than TNFi. However, these findings must be interpreted with caution given the limitations of this study and the low/very low certainty of the evidence.

**Systematic Review Registration:** https://www.crd.york.ac.uk/prospero/display_record.php?, identifier [CRD42020190838].

## 1 Introduction

Rheumatoid arthritis (RA) is a systemic inflammatory disease that affects the synovial fluid of joints, tendons, and some extra-articular sites (Tundia et al., 2016). Its estimated prevalence is 0.45% worldwide (Almutairi et al., 2021). The etiology of the disease is still unknown, but some studies point to the existence of an antigen that causes the synovial inflammatory process. In addition, there are risk factors such as genetics, heredity, hormones, environment, and habits and customs (Andrade and Dias, 2019).

Clinical Protocols and Therapeutic Guidelines indicate disease-modifying drugs (DMARD), starting with monotherapy with conventional synthetic DMARDs (csDMARDs) in first-line treatment, such as methotrexate. The use of biological DMARDs (bDMARDs) may be necessary in case of therapeutic failure or toxicity. This second class of drugs entails exceptionally high costs for patients, families, and healthcare systems (Coimbra De Oliveira, 2018).

The biologic agents have been highly effective and are comparable in reducing RA symptoms, slowing disease progression, and improving physical function (Donahue et al., 2008; Yun et al., 2016). However, because of the different immune-modulatory properties of specific drugs and drug classes, concerns have been raised about the risks of several potential adverse effects, including hospitalized infection, malignancy, congestive heart failure, and mortality, which could place a significant burden on patients and health care systems (Yun et al., 2016).

Administrative health databases are massive repositories of data collected in healthcare for various purposes, maintained in hospitals, health maintenance organizations, and health insurance organizations. Administrative databases may contain a variety of information such as medical claims for reimbursement, records of health services, medical procedures, prescriptions, diagnoses, and socioeconomic and demographic information. Therefore, data from administrative health databases may provide a sufficiently large and representative sample of subjects, contributing to meaningful, valid, and generalizable findings (Gavrielov-Yusim and Friger, 2014).

All over the world, there are databases of health information systems that have provided valuable information on rheumatic diseases and the use of biological medicines. Such data are used in pharmacovigilance and academic research, enabling the improvement of knowledge about the use of biological drugs. The constant improvement, referenced by a solid scientific framework, is built through multiple bases, increasing heterogeneity and size samples, hence the power of statistical analyses.

Despite the wide use of such databases along with clinical research, questions remain about possible risks associated with the use of medications, as well as the dimension of their adverse events (Donahue et al., 2008), requiring permanent surveillance of their use, especially in the treatment of RA (Desai et al., 2016; Harada et al., 2017; Dreyer et al., 2018). Therefore, this systematic review and meta-analysis aimed to assess the safety of biological therapy in patients with rheumatoid arthritis in observational studies using administrative health databases.

## 2 Methods

This systematic review and meta-analysis followed the Preferred Reporting Items for Systematic Reviews and Meta-analyses (PRISMA) Statement (Page et al., 2021). Before starting the literature search, the protocol for this systematic review was registered in the International Prospective Register of Systematic Review (PROSPERO) database (CRD42020190838).

### 2.1 Eligibility criteria

The PICOS structure was adopted to define the eligibility criteria. The population of interest (P) was patients with rheumatoid arthritis, the intervention (I) was the use of biological drugs (adalimumab, certolizumab pegol, etanercept, golimumab, infliximab, abatacept, rituximab, and tocilizumab), the comparator (C) was patients with rheumatoid arthritis unexposed to biological drugs or exposed to different drug classes, and the outcomes of interest (O) were adverse events and/or serious adverse events, and death.

Observational studies with administrative databases were eligible for inclusion. No language or date restrictions were applied. Clinical trials, review articles, case reports, case series, and animal studies were excluded.

### 2.2 Outcomes

The safety outcomes considered for inclusion in this systematic review and meta-analysis included adverse events (AEs) and/or serious adverse events (SAEs) such as infections (fungal, bacterial, and viral), tumors and cancer, cardiovascular events, and death.

### 2.3 Search strategy

Searches were performed in Embase, Lilacs (Virtual Health Library), MEDLINE (PubMed), MEDLINE and Epub Ahead of Print (Ovid), Scopus, and Web of Science Core Collection to identify studies that assessed the safety of biological therapy in patients with rheumatoid arthritis from inception to 21 October 2021. Moreover, gray literature sources (Catálogo de Teses e Dissertações da CAPES and specialized journals) were searched to identify studies that were not indexed in the databases but might be appropriate for inclusion in this systematic review.

Published articles and conference papers registered in these databases were identified using the terms “rheumatoid arthritis,” “adalimumab,” “certolizumab pegol,” “golimumab,” “infliximab,” “abatacept,” “rituximab,” “tocilizumab,” “biosimilar agent,” “hydroxychloroquine,” “methotrexate,” “salazosulfapyridine,” “administrative personnel,” “observational study,” and “cohort analysis” in Embase; “rheumatoid arthritis,” “adalimumab,” “certolizumab pegol,” “golimumab,” “infliximab,” “abatacept,” “rituximab,” “tocilizumab,” “antirheumatic agents,” “methotrexate,” “hydroxychloroquine,” “sulfasalazine,” “biosimilar pharmaceuticals,” “administrative personnel,” and “cohort studies” in Virtual Health Library; “rheumatoid arthritis,” “adalimumab,” “certolizumab pegol,” “golimumab,” “infliximab,” “abatacept,” “rituximab,” “tocilizumab,” “antirheumatic agents,” “methotrexate,” “hydroxychloroquine,” “sulfasalazine,” “biosimilar pharmaceuticals,” “administrative personnel,” and “cohort studies” in Pubmed; “rheumatoid arthritis,” “adalimumab,” “certolizumab pegol,” “golimumab,” “infliximab,” “abatacept,” “rituximab,” “tocilizumab,” “antirheumatic agents,” “methotrexate,” “hydroxychloroquine,” “sulfasalazine,” “biosimilar pharmaceuticals,” “administrative personnel,” and “cohort stud*” in Ovid, Scopus, and Web of Science. Search process details are presented in Supplementary Table S1.

### 2.4 Study selection and data extraction

Two reviewers (CCB and LG) independently screened articles’ titles and abstracts for potentially relevant articles using Rayyan (Ouzzani et al., 2016). Studies that met the inclusion criteria in the first screening had their eligibility confirmed by full reading. Articles that met all the inclusion criteria were included in the final review. A third reviewer (DBS) was referred to in cases of disagreement.

Two reviewers extracted the included studies’ details (MJQ and FCA). The extracted data include information related to authors, journal, publication year, country, sample size, safety outcomes, statistical analysis method (including statistical tests and measure of association with confidence intervals), and adjustment variables (confounders).

### 2.5 Methodological quality assessment

Two reviewers (CTC and MJQ) assessed the methodological quality of the included studies using the Newcastle-Ottawa Scale (NOS) (Wells et al., 2012). This tool has three domains with a score based on a star system, ranging from zero to nine stars: selection (four stars), comparability (two stars), and exposure or outcome of interest (three stars). Studies with a score of 0–3 stars were considered low-quality, those with a score of 4–6 stars were evaluated as moderate quality, and those which scored seven or more stars were classified as high-quality (Neal et al., 2019).

### 2.6 Statistical analysis

Data were extracted from eligible studies and arranged in a 2 × 2 table. The fixed or random-effects model was used to calculate risk ratios (RR) and 95% confidence intervals (95% CI), depending on the heterogeneity between the studies. Heterogeneity and consistency were evaluated by the I^2^ statistic and Cochran’s Q test (Higgins, 2003). A random-effects model was adopted when heterogeneity was verified (I^2^ > 50%; *p* < 0.10). The analysis was divided into five groups: tumor necrosis factor inhibitors (TNFi) versus non-TNFi; TNFi versus csDMARDs; bDMARDs versus csDMARDs; abatacept versus bDMARDs; and TNFi versus Janus kinase inhibitors (JAKi). A subgroup analysis by adverse event was conducted. Publication bias was assessed by visual inspection of the funnel plot and statistically using Egger’s tests. Analyses were carried out using R version 4.1.2 and the “meta” package version 4.13-0 (Balduzzi et al., 2019).

### 2.7 Assessment of the certainty of the evidence

The certainty of the evidence was rated using GRADEpro software (Grading of Recommendations, Assessment, Development and Evaluation). This system grades the quality of evidence at four levels—high, moderate, low, or very low—according to study design limitations, indirect evidence, inconsistency of results, inaccuracy of results, and the significant likelihood of publication bias (Schünemann et al., 2013).

## 3 Results

### 3.1 Selected studies

**FIGURE 1:**
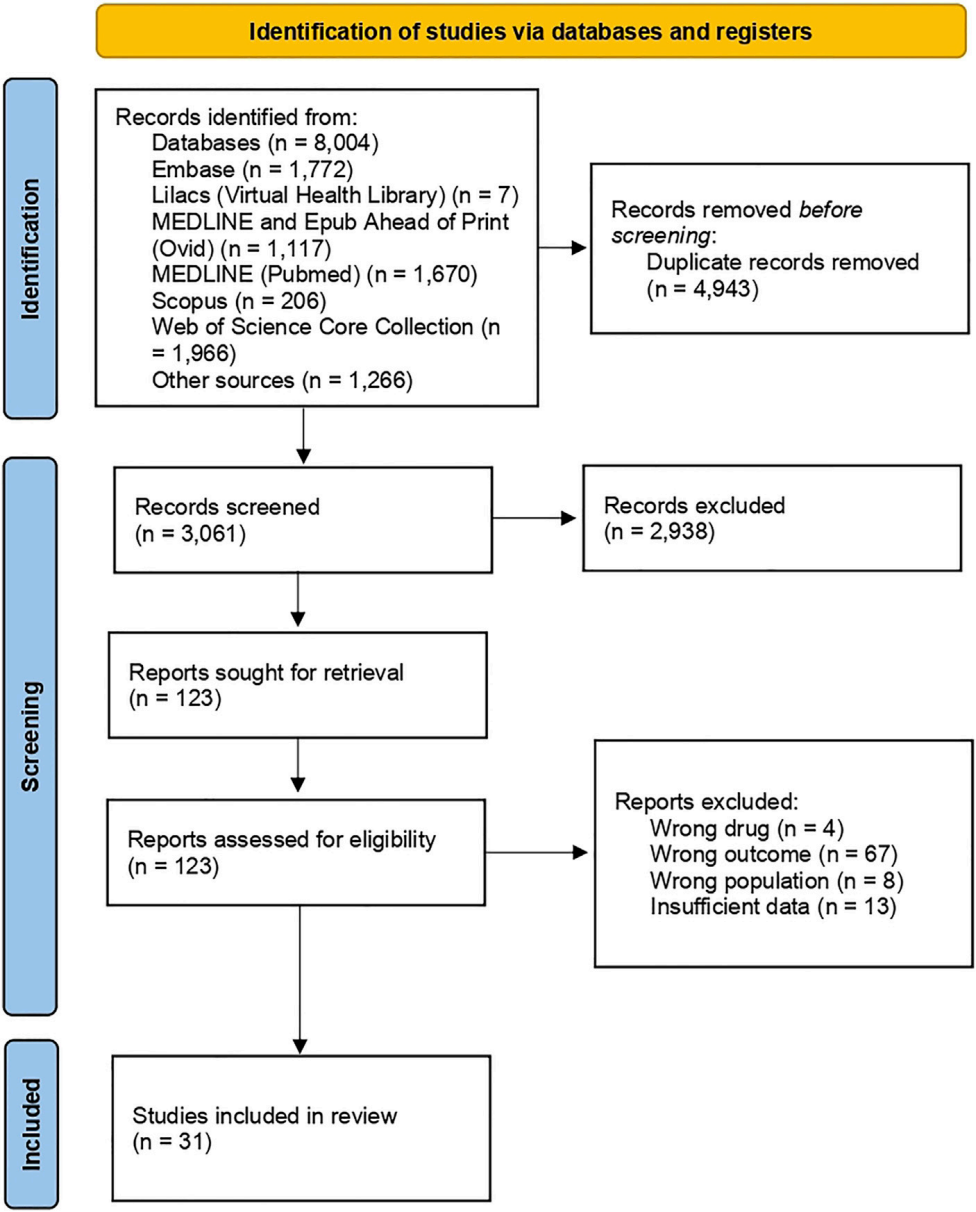
Flow chart of search results.

The initial search returned 8,004 studies, of which 4,943 were duplicates. After screening titles and abstracts, 123 studies were analyzed regarding inclusion criteria, and 92 were excluded. Subsequently, references of the included studies were manually searched to detect relevant articles, but none were identified. Studies were excluded due to the analysis of the wrong drug, outcome and population, and insufficient data (Figure 1). Details on the reasons and references excluded after the full reading are available in the Supplementary Material (Supplementary Table S2).

### 3.2 Study characteristics

Thirty-one studies were eligible for inclusion in the present systematic review; eleven population-based cohorts (Arkema et al., 2015; Raaschou et al., 2015; Mercer et al., 2015; Mercer et al., 2017; Desai et al., 2016; Low et al., 2017; Dreyer et al., 2018; Chen et al., 2020; Kim et al., 2020; Pettipher and Benitha, 2020; Hellgren et al., 2021), eight prospective (Listing et al., 2015; Richter et al., 2016; Meissner et al., 2017; Rutherford et al., 2018; Grøn et al., 2019; Grøn et al., 2020; Rahman et al., 2020; Ozen et al., 2021) and eight retrospective cohorts (Yun et al., 2014; 2016; Curtis et al., 2016; Zhang et al., 2016; Kim et al., 2017; Pawar et al., 2019; 2020; Patel et al., 2021), and four case-control studies (Harada et al., 2017; Sakai et al., 2018; de Germay et al., 2020; Chen et al., 2021), published from 2014 to 2021 (Supplementary Table S3).

A total of 1,039,398 rheumatoid arthritis patients were assessed. The mean age ranged between 46 and 78 years and most were women (60–100%). Mean disease duration was reported by thirteen studies and ranged between 3.4 and 16.5 years (Listing et al., 2015; Raaschou et al., 2015; Richter et al., 2016; Harada et al., 2017; Low et al., 2017; Meissner et al., 2017; Dreyer et al., 2018; Rutherford et al., 2018; Grøn et al., 2019; Rahman et al., 2020; Chen et al., 2021; Hellgren et al., 2021; Ozen et al., 2021). Among the thirteen studies which described mean disease activity, RA patients had moderate to high disease activity (Listing et al., 2015; Raaschou et al., 2015; Richter et al., 2016; Harada et al., 2017; Low et al., 2017; Meissner et al., 2017; Dreyer et al., 2018; Rutherford et al., 2018; Grøn et al., 2019; Kim et al., 2020; Pettipher and Benitha, 2020; Rahman et al., 2020; Hellgren et al., 2021) (Table 1).

**TABLE 1:** Characteristics of the included studies.

Study	Year	Country	Patients	Person-years	Number of events	Female (%)	Mean disease duration (years)	Mean disease activity	Outcome
Arkema	2014	Sweden	48,782	271,889	50	71.4 to 75.6	NR	NR	Tuberculosis
Chen	2020	United States	65,734	15,840	619	83.0 to 84.0	NR	NR	Hospitalized infection
Chen	2021	Taiwan	197,935	519,971	7,580	63.1	3.4	NR	Cardiovascular diseases
Curtis	2016	United States	63,102	40,507.4	2,264	79.7 to 83.7	NR	NR	Herpes zoster
Desai	2017	United States	7,222	9,918	370	75.0 to 79.0	NR	NR	Hypertension
Dreyer	2017	Denmark	1,678	3,686	108	70.3	10.0 to 16.0	DAS28: 3.4 to 5.1	Second malignant neoplasm
de Germay	2020	United States	15,846	NR	16,192	80.6 to 82.8	NR	NR	Cancer
Grøn	2019	Denmark and Sweden	8,987	10,873	639	76.0 to 81.0	7.0 to 11.0	DAS28: 4.7 to 5.1	Serious infection
Grøn	2020	Denmark	3,696	2,720	2,060	78.0	NR	NR	Infection
Harada	2017	Japan	1,987	6,753.5	43	81.5	6.0	DAS28: 4.2	Herpes zoster
Hellgren	2020	Sweden	71,645	450,828	392	NR	6.7	DAS28: 4.8	Lymphoma
Kim	2017	United States	40,119	22,046	125	81.7 to 84.7	NR	NR	Cardiovascular diseases
Kim	2020	Korea	996	NR	62	87.1	NR	DAS28 to ESR: 4.7	Hypertension
Listing	2015	Germany	8,908	31,378	463	77.3	10.3	DAS28: 5.3	Death
Low	2017	United Kingdom	14,258	65,973	252	59.5 to 78.0	6.0 to 11.0	DAS28: 5.3 to 6.6	Myocardial infarction
Meissner	2017	Germany	489	NR	166	74.8	9.7	DAS28: 5.1	Stroke
Mercer	2015	United Kingdom	15,016	64,221	563	73.0 to 76.0	NR	NR	Solid cancer
Mercer	2017	United Kingdom	15,298	114,599	114	74.0 to 76.0	NR	NR	Lymphoma
Ozen	2021	United States	18,754	94,781	1,801	79.4	14.2	NR	Cardiovascular diseases
Patel	2021	United States	30,439	NR	8,046	81.2 to 85.7	NR	NR	Infection
Pawar	2019	United States	141,869	42,148	1,773	81.7 to 83.1	NR	NR	Serious infection
Pawar	2020	United States	130,718	100,790	3,140	78.0	NR	NR	Serious infection
Pettipher and Benitha	2019	South Africa	4,830	8,205	96	67.0 to 71.0	NR	SDAI: 40.9 to 45.4	Tuberculosis
Raaschou	2014	Sweden	11,343	1,142	18	100.0	NR	NR	Recurrence of breast cancer
Rahman	2020	Canada	1,577	4,048	126	77.0 to 86.6	6.5 to 9.8	DAS28 to ESR: 4.4 to 5.7	Cancer, serious infections, herpes zoster, tuberculosis, and opportunistic infections
Richter	2016	Germany	917	NR	1,017	64.2 to 73.5	14.5 to 16.5	DAS28: 4.3 to 4.6	Serious infection, sepsis, and death
Rutherford	2018	United Kingdom	19,282	46,772	2,606	76.1 to 79.6	11.0 to 16.0	DAS28: 5.9 to 6.6	Serious infection
Sakai	2018	Japan	164	82,176	760	81.5	NR	NR	Herpes zoster
Yun	2015	United States	10,183	7,807	2,666	78.8 to 84.6	NR	NR	Hospitalized infection
Yun	2016	United States	23,784	16,576	2,530	83.9 to 88.7	NR	NR	Hospitalized infection
Zhang	2016	United States	47,193	74,662	585	85.0	NR	NR	Acute myocardial infarction

NR: not reported.

The 31 studies evaluated eleven different biological drugs, among them TNFi (etanercept, infliximab, adalimumab, certolizumab pegol, and golimumab), non-TNFi (rituximab, abatacept, tocilizumab, and anakinra), JAKi (tofacitinib), and csDMARDs (mainly methotrexate). Furthermore, the adverse events evaluated by the studies were cancer (solid cancer and lymphoma), cardiovascular events, infection, herpes zoster, tuberculosis, and death (Supplementary Table S3).

### 3.3 Quality of the included studies

According to the NOS, 27 studies were classified as high quality, of which seven were “nine stars” (Mercer et al., 2015, 2017; Zhang et al., 2016; Meissner et al., 2017; Pawar et al., 2019; Chen et al., 2020; Hellgren et al., 2021), fifteen were “eight stars” (Yun et al., 2014, 2016; Arkema et al., 2015; Listing et al., 2015; Richter et al., 2016; Desai et al., 2016; Kim et al., 2017; Low et al., 2017; Rutherford et al., 2018; Dreyer et al., 2018; Grøn et al., 2019; Grøn et al., 2020; Pawar et al., 2020; Chen et al., 2021; Ozen et al., 2021), and five were “seven stars” (Raaschou et al., 2015; Curtis et al., 2016; de Germay et al., 2020; Kim et al., 2020; Patel et al., 2021). Four studies were considered moderate quality, of which two scored “six stars” (Harada et al., 2017; Sakai et al., 2018), one “five stars” (Rahman et al., 2020), and one “four stars” (Pettipher and Benitha, 2020) (Supplementary Table S4).

### 3.4 Meta-analysis

#### 3.4.1 TNFi versus non-TNFi

**FIGURE 2:**
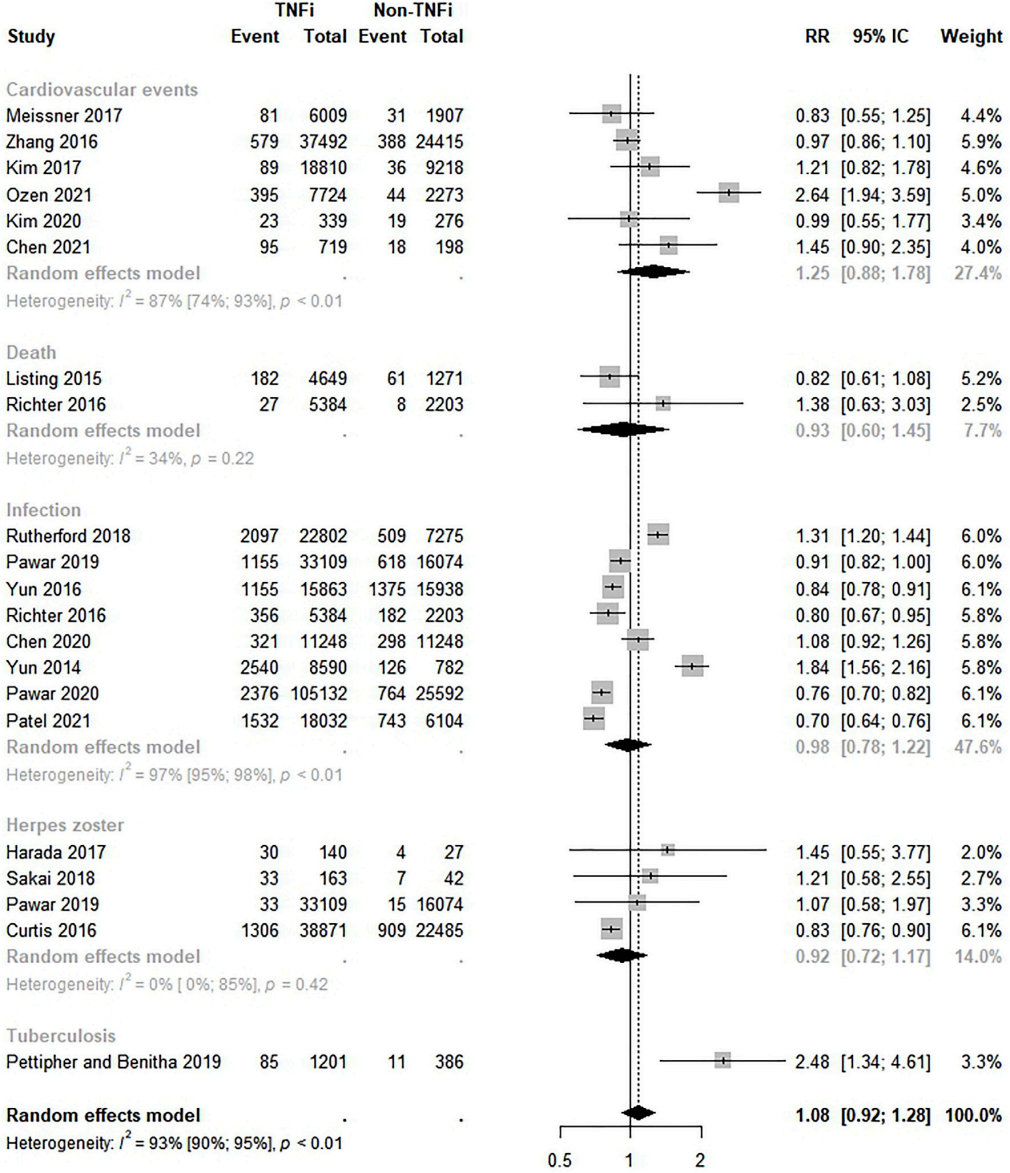
Comparative safety of TNF inhibitions and non-TNF inhibitions. TNFi: TNF inhibitions; non-TNFi: non-TNF inhibitions.

The safety of TNFi versus non-TNFi was assessed by 19 studies (Yun et al., 2014, 2016; Listing et al., 2015; Curtis et al., 2016; Richter et al., 2016; Zhang et al., 2016; Harada et al., 2017; Kim et al., 2017, 2020; Meissner et al., 2017; Rutherford et al., 2018; Sakai et al., 2018; Pawar et al., 2019, 2020; Chen et al., 2020, 2021; Pettipher and Benitha, 2020; Ozen et al., 2021; Patel et al., 2021). The meta-analysis revealed no significant differences in the safety of TNFi compared to non-TNFi (RR 1.08; 95% CI 0.92–1.28; *p* < 0.01; I^2^ = 93.0%). In the subgroup analysis, the risk of herpes zoster events was lower in the TNFi group (RR 0.92; 95% CI 0.72–1.17). In addition, subgroup analysis by safety outcome did not show a statistically significant higher risk of any outcomes among the TNFi (Figure 2), except for the tuberculosis event, which had a higher risk among TNFi; however, only one study was included. Visual inspection of the funnel plot indicated asymmetry, suggesting publication bias (Supplementary Figure S1). However, Egger’s test did not indicate publication bias (intercept = 2.44, *p* = 0.07).

#### 3.4.2 TNFi versus csDMARDs

**FIGURE 3:**
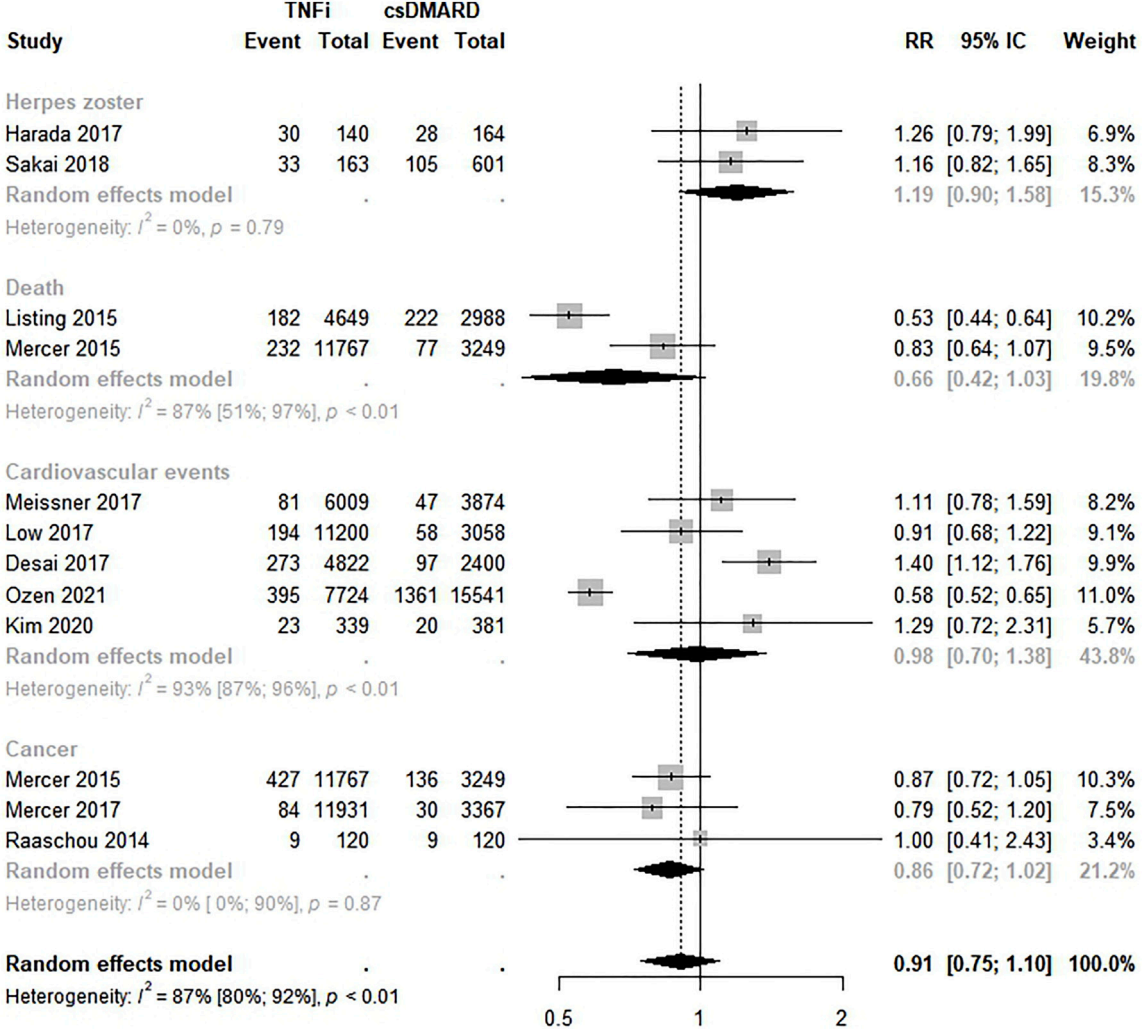
Comparative safety of TNF inhibitions and conventional disease-modifying anti-rheumatic drugs. TNFi: TNF inhibitions; cDMARD: conventional disease-modifying anti-rheumatic drugs.

Eleven studies evaluated the safety of TNFi compared to csDMARDs (Listing et al., 2015; Mercer et al., 2015; 2017; Raaschou et al., 2015; Desai et al., 2016; Harada et al., 2017; Low et al., 2017; Meissner et al., 2017; Sakai et al., 2018; Kim et al., 2020; Ozen et al., 2021). Overall, there was no significant difference in the safety of TNFi versus csDMARDs; however, a lower risk of events was found among TNFi (RR 0.91; 95% CI < 0.75–1.10; *p* < 0.01; I^2^ = 87.0%). Similarly, there were no significant differences between TNFi and csDMARDs by safety outcome (Figure 3). Funnel plot visual inspection suggested asymmetry (Supplementary Figure S2), and Egger’s test confirmed publication bias (intercept = 3.54, *p* = 0.02).

#### 3.4.3 bDMARDS versus csDMARDs

**FIGURE 4:**
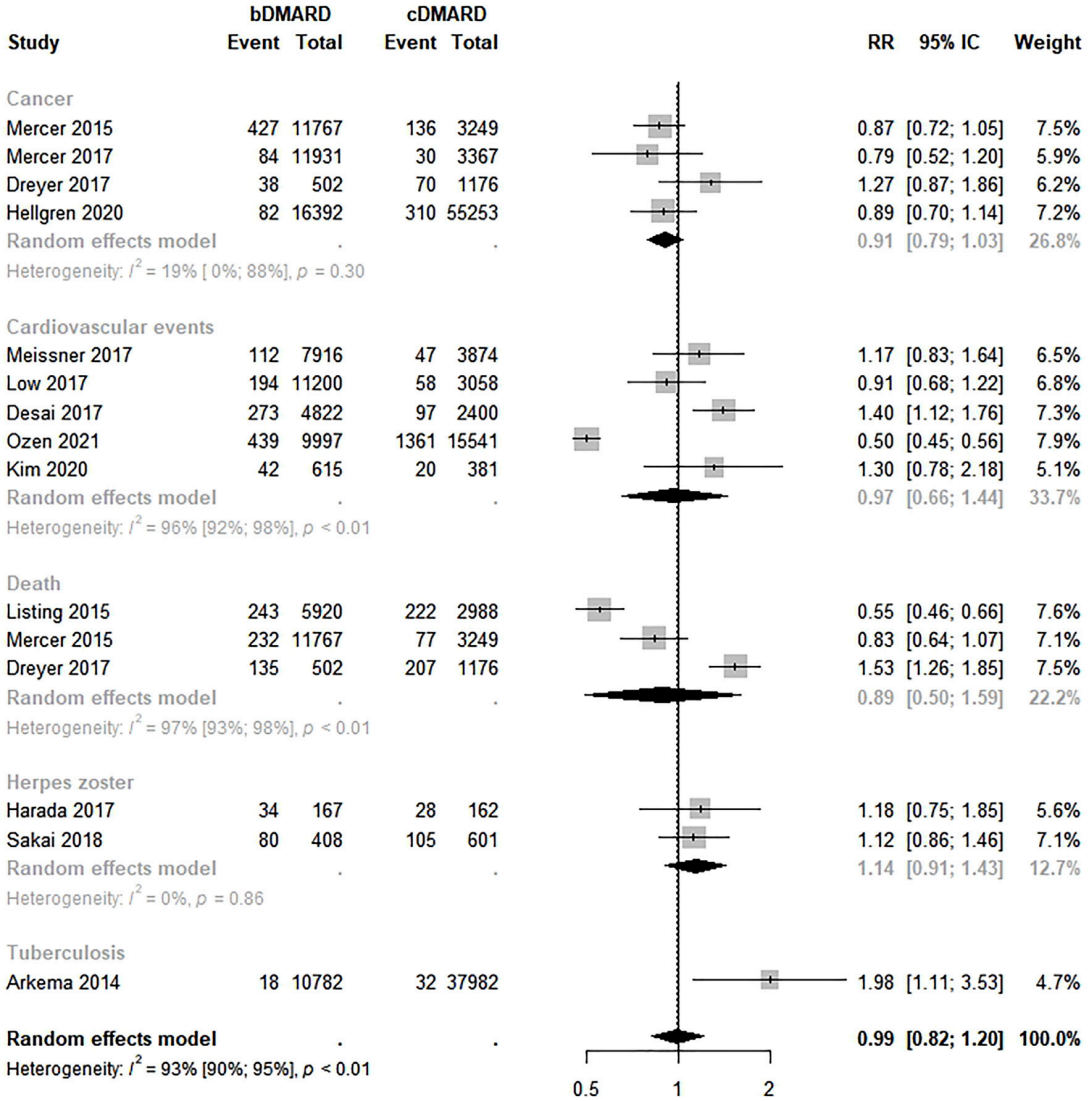
Comparative safety of biological disease-modifying anti-rheumatic drugs and conventional disease-modifying anti-rheumatic drugs. bDMARD: biological disease-modifying anti-rheumatic drugs; cDMARD: conventional disease-modifying anti-rheumatic drugs.

Thirteen studies estimated the safety of bDMARDs compared to csDMARDs (Arkema et al., 2015; Mercer et al., 2017; Listing et al., 2015; Mercer et al., 2015; Desai et al., 2016; Harada et al., 2017; Low et al., 2017; Meissner et al., 2017; Sakai et al., 2018; Dreyer et al., 2018; Kim et al., 2020; Ozen et al., 2021; Hellgren et al., 2021). No significant difference in the safety of these therapies was found (RR 0.99; 95% CI 0.82–1.20; *p* < 0.01; I^2^ = 93.0%). In the analysis by safety outcome, no statistically significant risk of any of the outcomes was observed (Figure 4). Funnel plot visualization suggests asymmetry (Supplementary Figure S3). The Egger’s test confirmed publication bias (intercept = 5.53, *p* = 0.01).

#### 3.4.4 Abatacept versus TNFi

**FIGURE 5:**
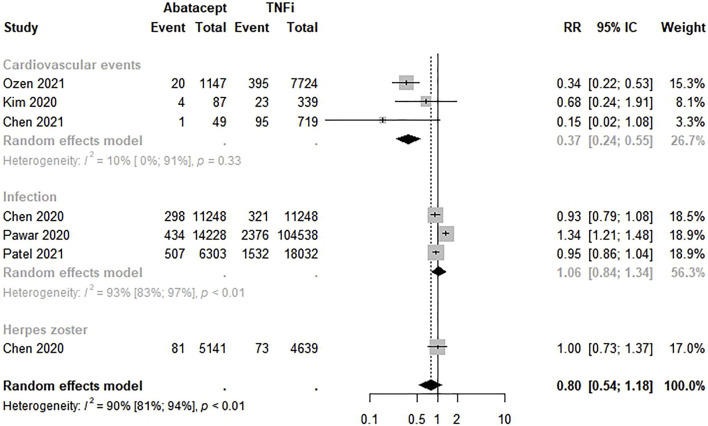
Comparative safety of abatacept and biological disease-modifying anti-rheumatic drugs. bDMARD: biological disease-modifying anti-rheumatic drugs.

The safety between abatacept and TNFi was evaluated by six studies (Chen et al., 2020, 2021; Kim et al., 2020; Pawar et al., 2020; Ozen et al., 2021; Patel et al., 2021). The meta-analysis showed a lower risk of adverse events, but there were no significant differences in the safety of abatacept compared to TNFi (RR 0.80; 95% CI 0.54–1.18; *p* < 0.01; I^2^ = 90.0%). However, a lower risk of cardiovascular events was found among RA patients who used abatacept rather than TNFi in the analysis by outcome measure (RR 0.37; 95% CI 0.24–0.55) (Figure 5).

#### 3.4.5 TNFi versus JAKi

**FIGURE 6:**
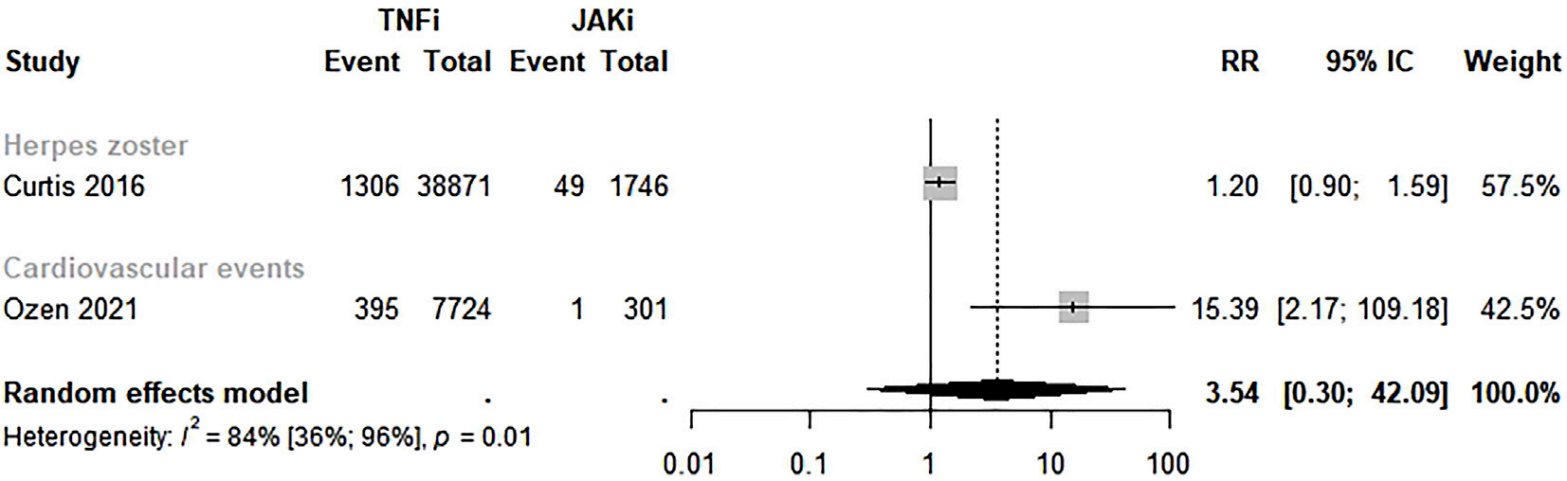
Comparative safety of TNF inhibitors and JAK inhibitors. TNFi: TNF inhibitors; JAKi: Janus Kinase inhibitors.

Only two studies evaluated the safety of TNFi versus JAKi (Curtis et al., 2016; Ozen et al., 2021). The meta-analysis revealed a higher risk of adverse events with no significant differences in the safety of TNFi compared to JAKi (RR 3.54; 95% CI 0.30–42.09; *p* = 0.01; I^2^ = 81.0%) (Figure 6).

### 3.5 Certainty of the evidence

The certainty of the evidence that contributed to the meta-analyses was low and very low due to the design of the studies, risk of bias, high heterogeneity between studies, low number of studies included in the analysis, and publication bias detected in some of the analyses (Supplementary Figures S4–S8). Therefore, this systematic review and meta-analysis results must be interpreted with caution.

## 4 Discussion

Our study estimated the safety of different drug classes of DMARDs in patients with rheumatoid arthritis based on observational studies with data from administrative databases. For studies with this type of data, it is important to confirm and expand the results obtained in clinical trials, as their homogeneity, the limited number of subjects, and relatively short follow-up time may limit the extrapolation of results. In addition, the increasing number of therapeutic alternatives require careful long-term follow-up to assess effectiveness and safety, which is only viable through observational studies, especially those from administrative health databases, taking into account the greatest amount of available data about patients’ medication and care (Suissa and Garbe, 2007; Ziemssen et al., 2017).

Our meta-analysis did not show significant differences in safety between TNFi versus non-TNFi, TNFi versus csDMARDs, bDMARDs versus csDMARDs, and TNFi versus JAKi for different safety outcomes, as cardiovascular events, death, infections, herpes zoster, cancer, and tuberculosis. However, a lower risk of cardiovascular events was found among RA patients who used abatacept in the analysis by outcome measure (RR 0.37; 95% CI 0.24–0.55) compared to TNFi.

RA and other inflammatory autoimmune rheumatic diseases are characterized by systemic inflammation, which contributes to atherosclerosis, endothelial dysfunction, plaque vulnerability, and atherothrombotic events, increasing the risk of cardiovascular disease in RA patients (Mackey et al., 2018). Nevertheless, cardiovascular disease is the leading cause of death and hospitalization among RA patients (Ozen et al., 2021).

Previous studies have reported a cardiovascular disease risk reduction in RA patients using DMARDs as hydroxychloroquine (Sharma et al., 2016), methotrexate (Micha et al., 2011), and TNFi (Low et al., 2017; Ozen et al., 2021). Nonetheless, despite several years and a considerable number of studies on cardiovascular events in patients with RA, there are still discrepant results. Even methotrexate, the most studied DMARD in the last 20 years, has not yet confirmed its cardioprotective action, hovering over the hypotheses of better control of disease activity or direct cardiovascular effect associated with the use of higher doses of the drug (Ozen et al., 2021). Therefore, our findings suggesting a 63% lower risk of these diseases among patients using abatacept compared to TNFi indicate a possible benefit for RA patients using this drug and must be further investigated.

Furthermore, evidence has shown an increased risk of certain types of solid cancers and lymphomas in people diagnosed with RA, with a strong association between the intensity of disease activity and inflammatory activity (Mercer et al., 2015; Hellgren et al., 2021). Although most patients from the studies included in the present systematic review had severe rheumatoid arthritis and poor prognosis, a higher risk of cancer was not observed in any of our meta-analyses. However, a systematic review and meta-analysis of 10 observational studies found an increased overall cancer (RR 1.13; 95% CI 1.02–1.24) and non-melanoma skin cancer risk (RR 1.26; 95% CI 1.09–1.45) among abatacept compared to csDMARDs or TNFi RA patients. Therefore, it is essential to closely monitor patients exposed to abatacept (Xie et al., 2020).

While high disease activity is a risk factor for infections in people with RA (Au et al., 2011; Mehta et al., 2019), biological therapy may increase the risk of serious infections due to its potent immunosuppressive effects. Furthermore, as biological drugs act on different cellular targets and cytokines, it can be hypothesized that the risk of infection may be different between them (Pawar et al., 2019), which brings concerns about clustered analysis of bDMARDs.

Our meta-analyses observed opposite effects between TNFi and non-TNFi regarding infection risk. Studies that used data from the Medicare, United States health insurances (Yun et al., 2016; Pawar et al., 2019, 2020; Patel et al., 2021), and the German biologics register RABBIT (Richter et al., 2016) presented a lower risk of infection in patients exposed to TNFi, while studies using data from the Medicare and Medicaid (Yun et al., 2014) and the British Society for Rheumatology Biologics Register (BSRBR-RA) (Rutherford et al., 2018) pointed to a higher risk of the outcome among TNFi-exposed subjects. These divergences may be related to differences in some patients’ characteristics, such as disease activity, previous exposure to biologic drugs, disease duration, comorbidities, age, and differences in follow-up time from baseline. Although the mechanisms of any risks remain unclear, the meta-analysis results showed no association between the comparative risk of TNFi drugs versus non-TNFi.

As stated before, RA is associated with an increased prevalence of several comorbidities, as cardiovascular disease, infection, malignancy, lung disease, and neuropsychiatric disease (Jeong et al., 2017). Nonetheless, it has also been observed that some comorbidities and external factors such as age, obesity, smoking, and dyslipidemia strongly influence the course of RA (Kłodziński and Wisłowska, 2018; Ozen et al., 2021). Therefore, these factors may affect this and other meta-analyses results since the studies adopted different techniques for adjusting those confounders and imputation of missing data.

In addition, the differences in the drugs selected to represent each class and the number of individuals taking them in each study should be highlighted. The individual effects observed for each drug may differ according to the number of individuals included in each study and the comparison with drugs or pharmacological groups that present different mechanisms of action. Still, some studies did not specify the number of individuals separately in the analysis by drug class, and some did not list the drugs in each category. We also highlight the underrepresentativeness of some biological medicines in the included studies, such as anakinra. This medicine was evaluated by only four of the included studies in this systematic review (Listing et al., 2015; de Germay et al., 2020; Hellgren et al., 2021; Ozen et al., 2021).

The concomitant use of other drugs not included in the analysis, such as glucocorticoids and immunosuppressive agents, may also interfere with our results. Unfortunately, however, most of the articles did not provide such information. Nevertheless, it is impossible to quantify its contribution to the observed effects even with this information due to the lack of supplementary data on dosage, time of exposure, and individual response to each medication or therapeutic regimen.

Furthermore, the use of prior biologics is widespread, and only a few studies verify the differences in the safety outcomes among biological-naïve and exposed (Arkema et al., 2015; Raaschou et al., 2015; Pettipher and Benitha, 2020). A population-based cohort with 48,782 RA patients from the Swedish Rheumatology Quality Register between 2002 and 2011 observed a higher risk of tuberculosis among biological-exposed compared with biological-naïve patients (HR 4.4; 95% CI 2.3–8.5) (Arkema et al., 2015). Pettipher and Benitha (2020), in a population-based cohort with data from 4,830 subjects from the South African Biologics Registry (SABIO) between 2008 and 2017, found a tuberculosis rate of 1,240 per 100,000 person-years for biologic users compared to 0 per 100,000 person-years among the biologic-naive cohort.

Moreover, TNFi-treated RA patients did not have a significantly higher risk of recurrent breast cancer than biologic-naïve patients (HR 1.1; 95% CI 0.4–2.8) in a population-based cohort with 11,343 subjects from the Swedish biologics register (ARTIS) between 2001 and 2010 (Raaschou et al., 2015).

Taking the disability-adjusted life years (DALYs) WHO indicator into account, which combines years of life lost to premature mortality (YLLs) and years of healthy life lost due to disability (YLDs), the systematic analysis of the Global Burden of Disease Study from 2017 showed almost 20 million prevalent cases of RA in that year, accounting for 1.2 million incident cases that resulted in 3.4 million disability-adjusted life years (DALYs) (Safiri et al., 2020). Based on the available evidence, it would not be reckless to say that the adverse effects associated with the medications can count as an adjuvant on time of healthy life lost due to disability.

Our results reassure the need for further post-market long-term studies for biological drugs. In this way, the best therapeutic choices can be ensured for patients with RA, given the severity of adverse effects of the drug therapy, aiming to improve their quality of life and prevent premature mortality related to RA.

### 4.1 Strengths and limitations

Our study has important strengths and limitations. Strengths include using a validated scale to assess individual studies’ methodological quality, evaluating the evidence’s certainty, and using random-effects meta-analysis to deal with the heterogeneity between studies. Furthermore, we contacted some authors to obtain sufficient data to perform the meta-analysis.

The high heterogeneity between studies, which persisted after subgroup analysis, was a limitation of the present study. Several factors could justify this, such as RA severity and prognosis differences, and some population characteristics.

Furthermore, the type of analysis used cannot treat confounders such as age, gender, ethnicity, level of education, work, type of health insurance, BMI, smoking, comorbidity, hypertension, diabetes, and use of drugs that can influence the outcome, such as statins, aspirin, NSAIDs, and the imputations made in several studies.

An important limitation is that some studies differ in the moment of drug exposure for the outcome. Therefore, experienced and naïve, prevalent, and incident individuals were included in the meta-analysis. Also, as the included studies followed patients with different pharmacological treatments at different times, a follow-up time bias cannot be discarded. These differences may influence the development of adverse events, such as cancer. Also, RA patients in non-TNFi therapy usually have a longer disease duration than those using TNFi and csDMARDs, which may impact and confound these meta-analyses results.

It is important to state that nowadays, RA patients tend to be exposed to more biological agents, relying on cumulative exposure to biologics, making it impossible to differentiate the results of current therapy from those of previous therapies. Besides, we could not analyze the safety outcomes by comparing biological-naïve and biologic-experienced patients due to the lack of studies making such comparisons. Also, some studies presented short baseline periods, which may introduce a misclassification bias in these studies.

There is the possibility of overlapping in some of the cohorts included, mainly those using data from Medicare. Overlap is a problem of precision related to sampling, so overlapping cohorts in systematic reviews may overstate sample size and the number of events, falsely leading to greater precision in the analysis (Lunny et al., 2021). Nonetheless, these cohort studies generally compared different drugs and outcomes, which probably reduced this effect in the present systematic review and meta-analysis.

Even though the prevalence of RA is considerably higher in older people, there are studies with only individuals over 65, such as those based on Medicare data (Yun et al., 2016; Zhang et al., 2016; Patel et al., 2021), which may influence our results. In addition, the use of health insurance databases can unbalance the results by selecting patients with higher earnings and better access to care.

Also, a low number of studies were included in the meta-analyses of abatacept versus TNFi and TNFi versus JAKi, which may be related to our search strategies when we chose to specify the name of each drug instead of including direct terms. Furthermore, the inclusion of low number of studies in meta-analysis may result in findings by chance. Nonetheless, meta-analyses with a small number of studies present valid results (Herbison et al., 2011). Finally, a small number of studies for these analyses excluded the possibility of publication bias analysis. However, it should be noted that the interpretation of graph asymmetry is subjective and interpretation errors may occur (Sterne et al., 2004).

The publication bias found in studies that evaluated TNFi versus csDMARDs and bDMARDS versus csDMARDs is probably associated with the eligibility criteria adopted, including only observational studies with administrative databases. Also, the inclusion of mesh terms related to the study design on the search strategy may have an impact on its sensitivity.

In summary, the present study suggests a decreased risk of cardiovascular events among abatacept users compared to TNFi users. In contrast, no significant differences in cardiovascular events, death, infections, herpes zoster, cancer, and tuberculosis were found between TNFi compared to non-TNFi, TNFi compared to csDMARDs, bDMARDs compared to csDMARDs, and TNFi compared to JAKi. Nonetheless, these data should be interpreted with caution given the limitations previously stated and the low/very low certainty of the evidence according to the GRADE. Therefore, further studies using administrative databases and longer follow-up times are needed to confirm our findings.

## Data Availability

The original contributions presented in the study are included in the article/Supplementary Material; further inquiries can be directed to the corresponding author.
