# Supplementary material for: Safety of biological therapy in patients with rheumatoid arthritis in administrative health databases: A systematic review and meta-analysis
Source: Front Pharmacol. 2022 Aug 11;13:928471. doi: 10.3389/fphar.2022.928471 (PMC9407686; doi:10.3389/fphar.2022.928471)
Supplement: Supplementary file 1 [file DataSheet1.docx]

***Supplementary Material***

**Contents**

**Table S1.** Search strategy by database.

**Table S2.** List of studies excluded after full reading.

**Table S3.** Additional characteristics of the included studies.

**Table S4.** Methodological quality assessment according to the Newcastle-Ottawa Scale.

**Figure S1.** Funnel plot of studies that compared the safety of TNFi versus non-TNFi.

**Figure S2.** Funnel plot of studies that compared the safety of TNFi versus csDMARDs.

**Figure S3.** Funnel plot of studies that compared the safety of bDMARDs versus csDMARDs.

**Figure S4.** GRADE assessment of the methodological quality of studies that evaluated TNFi versus non-TNFi.

**Figure S5.** GRADE assessment of the methodological quality of studies that evaluated TNFi versus csDMARDs.

**Figure S6.** GRADE assessment of the methodological quality of studies that evaluated bDMARDs versus csDMARDs.

**Figure S7.** GRADE assessment of the methodological quality of studies that evaluated Abatacept versus TNFi.

**Figure S8.** GRADE assessment of the methodological quality of studies that evaluated TNFi versus JAKi.

**Table S1.** Search strategy by database.

| **Database  (seach date: 21/10/2021)** | **Search terms** |
| --- | --- |
|  |  |
| Embase  (1999 - 2021) | 'rheumatoid arthritis'/exp AND ('adalimumab'/exp OR 'certolizumab pegol'/exp OR 'golimumab'/exp OR 'infliximab'/exp OR 'abatacept'/exp OR 'rituximab'/exp OR 'tocilizumab'/exp) AND ('biosimilar agent'/exp OR 'hydroxychloroquine'/exp OR 'methotrexate'/exp OR 'salazosulfapyridine'/exp) AND ('administrative personnel'/exp OR 'observational study'/exp OR 'cohort analysis'/exp) |
| Lilacs (via Virtual Health Library)  (2010 - 2021) | tw:((rheumatoid arthritis AND (((((((((((((((adalimumab) OR certolizumab pegol) OR golimumab) OR infliximab) OR abatacept) OR rituximab) OR tocilizumab) OR antirheumatic agents)) AND ((methotrexate) OR hydroxychloroquine) OR sulfasalazine) OR biosimilar pharmaceuticals) OR biosimilar) OR biosimilars) OR biosimilarity) OR follow on biologics)) AND ((administrative personnel) OR (cohort studies) OR (cohort study) OR (studies, cohort) OR (study, cohort) OR (concurrent studies) OR (studies, concurrent) OR (concurrent study) OR (study, concurrent) OR (historical cohort studies) OR (studies, historical cohort) OR (cohort studies, historical) OR (cohort study, historical) OR (historical cohort study) OR (study, historical cohort) OR (analysis, cohort) OR (analysis, cohort) OR (cohort analyses) OR (cohort analysis) OR (closed cohort studies) OR (cohort studies, closed) OR (closed cohort study) OR (cohort study, closed) OR (study, closed cohort) OR (studies, closed cohort) OR (incidence studies) OR (incidence study) OR (studies, incidence) OR (study, incidence) OR (cohort studies) OR (cohort) OR (cohort analysis) OR (cohort study) OR (prospective cohort) OR (retrospective cohort) OR (retrospective cohort study) OR (prospective cohort study) OR (follow-up studies) OR (follow up studies) OR (follow-up study) OR (studies, follow-up) OR (study, follow-up) OR followup studies OR (followup study) OR (studies, followup) OR (study, followup) OR (epidemiologic studies OR retrospective studies OR longitudinal studies OR prospective studies))) AND ( db:("LILACS")) |
| MEDLINE and Epub Ahead of Print  (via Ovid)  (2014 - 2021) | (rheumatoid arthritis) AND (adalimumab OR certolizumab pegol OR golimumab OR infliximab OR abatacept OR rituximab OR tocilizumab OR antirheumatic agents) AND (methotrexate OR hydroxychloroquine OR sulfasalazine OR biosimilar pharmaceuticals OR biosimilar* OR follow on biologics) AND (administrative personnel OR cohort Stud* OR concurrent stud* OR historical cohort stud* OR cohort anal* OR closed cohort stud* OR incidence stud* OR prospective cohort OR retrospective cohort OR retrospective cohort study OR prospective cohort study OR follow up stud* OR followup stud* OR follow-up stud* OR Epidemiologic Stud* OR retrospective stud* OR Longitudinal Stud* OR Prospective Stud*) NOT (review) |
| MEDLINE (via Pubmed)  (1983 - 2021) | ("Arthritis, rheumatoid"[MeSH Terms] AND (((((((((((((((((("adalimumab"[MeSH Terms]) OR "certolizumab pegol" [MeSH Terms]) OR "golimumab"[Supplementary Concept] OR "golimumab"[All Fields]) OR "golimumab s"[All Fields]) OR "infliximab" [MeSH Terms]) OR "abatacept" [MeSH Terms]) OR "rituximab" [MeSH Terms]) OR "tocilizumab" [Supplementary Concept]) OR "tocilizumab" [All Fields]) OR "antirheumatic agents"[MeSH Terms])) AND (("methotrexate" [MeSH Terms]) OR "hydroxychloroquine" [MeSH Terms]) OR "sulfasalazine" [MeSH Terms]) OR "biosimilar pharmaceuticals"[MeSH Terms]) OR ("biosimilar"[All Fields] AND "pharmaceuticals"[All Fields]) OR "biosimilar pharmaceuticals"[All Fields]) OR "biosimilar"[All Fields]) OR "biosimilars"[All Fields]) OR "biosimilarity"[All Fields]) OR "follow on biologics"[All Fields])) AND (("administrative personnel"[MeSH Terms]) OR ("Cohort Studies"[Mesh]) OR (cohort study) OR (studies, cohort) OR (study, cohort) OR (concurrent studies) OR (studies, concurrent) OR (concurrent study) OR (study, concurrent) OR (historical cohort studies) OR (studies, historical cohort) OR (cohort studies, historical) OR (cohort study, historical) OR (historical cohort study) OR (study, historical cohort) OR (analysis, cohort) OR (analysis, cohort) OR (cohort analyses) OR (cohort analysis) OR (closed cohort studies) OR (cohort studies, closed) OR (closed cohort study) OR (cohort study, closed) OR (study, closed cohort) OR (studies, closed cohort) OR (incidence studies) OR (incidence study) OR (studies, incidence) OR (study, incidence) OR (cohort studies) OR (cohort) OR (cohort analysis) OR (cohort study) OR (prospective cohort) OR (retrospective cohort) OR (retrospective cohort study) OR (prospective cohort study) OR ("Follow-Up Studies"[Mesh]) OR (follow up studies) OR (follow-up study) OR (studies, follow-up) OR (study, follow-up) OR followup studies OR (followup study) OR (studies, followup) OR (study, followup) OR ("Epidemiologic Studies"[Mesh] OR "Retrospective Studies"[Mesh] OR "Longitudinal Studies"[Mesh] OR "Prospective Studies"[Mesh])) |
| Scopus  (2006 - 2021) | ALL(rheumatoid arthritis) AND ALL(adalimumab OR certolizumab pegol OR golimumab OR infliximab OR abatacept OR rituximab OR tocilizumab OR antirheumatic agents) AND ALL(methotrexate OR hydroxychloroquine OR sulfasalazine OR biosimilar pharmaceuticals OR biosimilar* OR follow on biologics) AND ALL(administrative personnel OR cohort Stud* OR concurrent stud* OR historical cohort stud* OR cohort anal* OR closed cohort stud* OR incidence stud* OR prospective cohort OR retrospective cohort OR retrospective cohort study OR prospective cohort study OR follow up stud* OR followup stud* OR follow-up stud* OR Epidemiologic Stud* OR retrospective stud* OR Longitudinal Stud* OR Prospective Stud*) |
| Web of Science Core Collection  (2017 - 2021) | ALL=(rheumatoid arthritis) AND ALL=(adalimumab OR certolizumab pegol OR golimumab OR infliximab OR abatacept OR rituximab OR tocilizumab OR antirheumatic agents) AND ALL=(methotrexate OR hydroxychloroquine OR sulfasalazine OR biosimilar pharmaceuticals OR biosimilar* OR follow on biologics) AND ALL=(administrative personnel OR cohort Stud* OR concurrent stud* OR historical cohort stud* OR cohort anal* OR closed cohort stud* OR incidence stud* OR prospective cohort OR retrospective cohort OR retrospective cohort study OR prospective cohort study OR follow up stud* OR followup stud* OR follow-up stud* OR Epidemiologic Stud* OR retrospective stud* OR Longitudinal Stud* OR Prospective Stud*) |

**Table S2.** List of studies excluded after full reading.

| **Reasons** | **References** |
| --- | --- |
| **Wrong drug** | 1. Guidelli, G. M., Viapiana, O., Luciano, N., De Santis, M., Boffini, N., Quartuccio, L., et al. (2021). Efficacy and safety of baricitinib in 446 patients with rheumatoid arthritis: a real-life multicentre study. Clin. Exp. Rheumatol. 39, 868–873. 2. Kang, E. H., Jin, Y., Tong, A. Y., Desai, R. J., and Kim, S. C. (2020). Risk of Serious Infection Among Initiators of Tumor Necrosis Factor Inhibitors Plus Methotrexate Versus Triple Therapy for Rheumatoid Arthritis: A Cohort Study. Arthritis Care Res. (Hoboken). 72, 1383–1391. doi:10.1002/acr.24038. 3. Krasselt, M., Baerwald, C., Petros, S., and Seifert, O. (2021). Mortality of Sepsis in Patients With Rheumatoid Arthritis: A Single-Center Retrospective Analysis and Comparison With a Control Group. J. Intensive Care Med. 36, 766–774. doi:10.1177/0885066620917588. 4. Seror, R., Lafourcade, A., De-Rycke, Y., Fautrel, B., Mariette, X., and Tubach, F. (2020). Risk of malignancies associated with cs DMARDs in rheumatoid arthritis: Comparison with general population and biologic treated patients (analysis of a national claim database). Ann. Rheum. Dis. 79, 291. |
| **Wrong outcome** | 1. Acurcio, F. A., Machado, M. A. A. A., Moura, C. S., Ferre, F., Guerra, A. A., Andrade, E. I. G. G., et al. (2016). Medication Persistence of Disease-Modifying Antirheumatic Drugs and Anti-Tumor Necrosis Factor Agents in a Cohort of Patients With Rheumatoid Arthritis in Brazil. Arthritis Care Res. (Hoboken). 68, 1489–1496. doi:10.1002/acr.22840. 2. Aladul, M. I., Fitzpatrick, R. W., and Chapman, S. R. (2017). Impact of Infliximab and Etanercept Biosimilars on Biological Disease-Modifying Antirheumatic Drugs Utilisation and NHS Budget in the UK. BioDrugs 31, 533–544. doi:10.1007/s40259-017-0252-3. 3. Asai, S., Kojima, T., Oguchi, T., Kaneko, A., Hirano, Y., Yabe, Y., et al. (2015). Effects of Concomitant Methotrexate on Large Joint Replacement in Patients With Rheumatoid Arthritis Treated With Tumor Necrosis Factor Inhibitors: A Multicenter Retrospective Cohort Study in Japan. Arthritis Care Res. (Hoboken). 67, 1363–1370. doi:10.1002/acr.22596. 4. Barbulescu, A., Delcoigne, B., Askling, J., and Frisell, T. (2020). Gastrointestinal perforations in patients with rheumatoid arthritis treated with biological disease-modifying antirheumatic drugs in Sweden: a nationwide cohort study. RMD Open 6, e001201. doi:10.1136/rmdopen-2020-001201. 5. Bechman, K., Oke, A., Yates, M., Norton, S., Dennison, E., Cope, A. P., et al. (2020). Is background methotrexate advantageous in extending TNF inhibitor drug survival in elderly patients with rheumatoid arthritis? An analysis of the British Society for Rheumatology Biologics Register. Rheumatology 59, 2563–2571. doi:10.1093/rheumatology/kez671. 6. Bird, P., Littlejohn, G., Butcher, B., Smith, T., da Fonseca Pereira, C., Witcombe, D., et al. (2020). Real-world evaluation of effectiveness, persistence, and usage patterns of tofacitinib in treatment of rheumatoid arthritis in Australia. Clin. Rheumatol. 39, 2545–2551. doi:10.1007/s10067-020-05021-7. 7. Chatzidionysiou, K., Askling, J., Eriksson, J., Kristensen, L. E., and van Vollenhoven, R. (2015). Effectiveness of TNF inhibitor switch in RA: results from the national Swedish register. Ann. Rheum. Dis. 74, 890–896. doi:10.1136/annrheumdis-2013-204714. 8. Chen, J.-F., Hsu, C.-Y., Yu, S.-F., Ko, C.-H., Chiu, W.-C., Lai, H.-M., et al. (2020). The impact of long-term biologics/target therapy on bone mineral density in rheumatoid arthritis: a propensity score-matched analysis. Rheumatology 59, 2471–2480. doi:10.1093/rheumatology/kez655. 9. Choi, S., Ghang, B., Jeong, S., Choi, D., Lee, J. S., Park, S. M., et al. (2021). Association of first, second, and third-line bDMARDs and tsDMARD with drug survival among seropositive rheumatoid arthritis patients: Cohort study in A real world setting. Semin. Arthritis Rheum. 51, 685–691. doi:10.1016/j.semarthrit.2021.06.002. 10. Choy, E., Groves, L., Sugrue, D., Hurst, M., Houghton, J., Venkatachalam, S., et al. (2021). Outcomes in rheumatoid arthritis patients treated with abatacept: a UK multi-centre observational study. BMC Rheumatol. 5, 3. doi:10.1186/s41927-020-00173-0. 11. Codreanu, C., Popescu, C. C., Mogoșan, C., Enache, L., Daia, S., Ionescu, R., et al. (2019). Efficacy and safety of original and biosimilar etanercept (SB4) in active rheumatoid arthritis – A comparison in a real-world national cohort. Biologicals 62, 27–32. doi:10.1016/j.biologicals.2019.10.009. 12. Crane, M. M., Juneja, M., Allen, J., Kurrasch, R. H., Chu, M. E., Quattrocchi, E., et al. (2015). Epidemiology and Treatment of New-Onset and Established Rheumatoid Arthritis in an Insured US Population. Arthritis Care Res. (Hoboken). 67, 1646–1655. doi:10.1002/acr.22646. 13. Croiteru, A., Lidar, M., Reitblat, T., Zisman, D., Balbir-Gurman, A., Meshiach, T., et al. (2019). Real life retention of tofacitinib in patients with rheumatoid arthritis. Arthritis Rheumatol. 71, 2443–2444. 14. Curtis, J. R., Chakravarty, S. D., Black, S., Kafka, S., Xu, S., Langholff, W., et al. (2021). Incidence of Infusion Reactions and Clinical Effectiveness of Intravenous Golimumab Versus Infliximab in Patients with Rheumatoid Arthritis: The Real-World AWARE Study. Rheumatol. Ther. 8, 1551–1563. doi:10.1007/s40744-021-00354-4. 15. Curtis, J. R., Chastek, B., Becker, L., Quach, C., Harrison, D. J., Yun, H., et al. (2015). Cost and Effectiveness of Biologics for Rheumatoid Arthritis in a Commercially Insured Population. J. Manag. Care Spec. Pharm. 21, 318–329. doi:10.18553/jmcp.2015.21.4.318. 16. Dormuth, C. R., Fisher, A., Hudson, M., Austin, P. C., Ernst, P., Bresee, L., et al. (2021). Impact of using concomitant conventional DMARDs on adherence to biologic DMARD treatment in rheumatoid arthritis: Multi-centre, population-based cohort study. Semin. Arthritis Rheum. 51, 1291–1299. doi:10.1016/J.SEMARTHRIT.2021.08.002. 17. Ebina, K., Hirano, T., Maeda, Y., Yamamoto, W., Hashimoto, M., Murata, K., et al. (2020a). Drug retention of 7 biologics and tofacitinib in biologics-naïve and biologics-switched patients with rheumatoid arthritis: the ANSWER cohort study. Arthritis Res. Ther. 22, 142. doi:10.1186/s13075-020-02232-w. 18. Ebina, K., Hirano, T., Maeda, Y., Yamamoto, W., Hashimoto, M., Murata, K., et al. (2020b). Drug retention of secondary biologics or JAK inhibitors after tocilizumab or abatacept failure as first biologics in patients with rheumatoid arthritis -the ANSWER cohort study-. Clin. Rheumatol. 39, 2563–2572. doi:10.1007/s10067-020-05015-5. 19. Favalli, E. G., Becciolini, A., Biggioggero, M., Bertoldi, I., Crotti, C., Raimondo, M. G., et al. (2018). The role of concomitant methotrexate dosage and maintenance over time in the therapy of rheumatoid arthritis patients treated with adalimumab or etanercept: retrospective analysis of a local registry. Drug Des. Devel. Ther. Volume 12, 1421–1429. doi:10.2147/DDDT.S162286. 20. Gendelman, O., Weitzman, D., Rosenberg, V., Shalev, V., Chodick, G., and Amital, H. (2018). Characterization of adherence and persistence profile in a real-life population of patients treated with adalimumab. Br. J. Clin. Pharmacol. 84, 786–795. doi:10.1111/bcp.13494. 21. George, M. D., Baker, J. F., and Ogdie, A. (2020). Comparative Persistence of Methotrexate and Tumor Necrosis Factor Inhibitors in Rheumatoid Arthritis, Psoriatic Arthritis, and Ankylosing Spondylitis. J. Rheumatol. 47, 826–834. doi:10.3899/jrheum.190299. 22. Gharaibeh, M., Bonafede, M., McMorrow, D., Hernandez, E. J. M., and Stolshek, B. S. (2020). Effectiveness and Costs Among Rheumatoid Arthritis Patients Treated with Targeted Immunomodulators Using Real-World U.S. Data. J. Manag. Care Spec. Pharm. 26, 1039–1049. doi:10.18553/jmcp.2020.26.8.1039. 23. Giraud, E. L., Jessurun, N. T., van Hunsel, F. P. A. M., van Puijenbroek, E. P., van Tubergen, A., Ten Klooster, P. M., et al. (2020). Frequency of real-world reported adverse drug reactions in rheumatoid arthritis patients. Expert Opin. Drug Saf. 19, 1617–1624. doi:10.1080/14740338.2020.1830058. 24. Glintborg, B., Loft, A. G., Omerovic, E., Hendricks, O., Linauskas, A., Espesen, J., et al. (2019). To switch or not to switch: results of a nationwide guideline of mandatory switching from originator to biosimilar etanercept. One-year treatment outcomes in 2061 patients with inflammatory arthritis from the DANBIO registry. Ann. Rheum. Dis. 78, 192–200. doi:10.1136/annrheumdis-2018-213474. 25. Harrold, L. R., Reed, G. W., Kremer, J. M., Curtis, J. R., Solomon, D. H., Hochberg, M. C., et al. (2015). The comparative effectiveness of abatacept versus anti-tumour necrosis factor switching for rheumatoid arthritis patients previously treated with an anti-tumour necrosis factor. Ann. Rheum. Dis. 74, 430–436. 26. Helliwell, P. S., and Taylor, W. J. (2008). Treatment of psoriatic arthritis and rheumatoid arthritis with disease modifying drugs -- comparison of drugs and adverse reactions. J. Rheumatol. 35, 472–476. 27. Iacono, D., Pantano, I., Birra, D., Scalise, G., Coscia, M. A., Messiniti, V., et al. (2020). Retention rate of abatacept monotherapy in an italian multicentric rheumatoid arthritis cohort. Ann. Rheum. Dis. 79, 1456–1457. 28. Izumi, K., Kaneko, Y., Yasuoka, H., Seta, N., Kameda, H., Kuwana, M., et al. (2015). Tocilizumab is clinically, functionally, and radiographically effective and safe either with or without low-dose methotrexate in active rheumatoid arthritis patients with inadequate responses to DMARDs and/or TNF inhibitors: A single-center retrospective. Mod. Rheumatol. 25, 31–37. doi:10.3109/14397595.2014.897793. 29. Kihara, M., Davies, R., Kearsley-Fleet, L., Watson, K. D., Lunt, M., Symmons, D. P. M., et al. (2017). Use and effectiveness of tocilizumab among patients with rheumatoid arthritis: an observational study from the British Society for Rheumatology Biologics Register for rheumatoid arthritis. Clin. Rheumatol. 36, 241–250. 30. Kim, G., Barner, J. C., Rascati, K., and Richards, K. (2016). Examining Time to Initiation of Biologic Disease-modifying Antirheumatic Drugs and Medication Adherence and Persistence Among Texas Medicaid Recipients With Rheumatoid Arthritis. Clin. Ther. 38, 646–654. doi:10.1016/j.clinthera.2016.01.022. 31. Klink, A., Han, X., Lobo, F., Szymialis, R., Lam, J., and Feinberg, B. (2020). Clinical benefits reported in ample trial observed in a real-world (RW) cohort of us rheumatoid arthritis (RA) patients. Ann. Rheum. Dis. 79, 626–627. 32. Krause, D., Krause, C., Rudolf, H., Baraliakos, X., Braun, J., and Schmitz, E. (2021). Dose tapering of biologic agents in patients with rheumatoid arthritis—results from a cohort study in Germany. Clin. Rheumatol. 40, 887–893. doi:10.1007/s10067-020-05316-9. 33. Lauper, K., Nordström, D. C., Pavelka, K., Hernández, M. V., Kvien, T. K., Kristianslund, E. K., et al. (2018). Comparative effectiveness of tocilizumab versus TNF inhibitors as monotherapy or in combination with conventional synthetic disease-modifying antirheumatic drugs in patients with rheumatoid arthritis after the use of at least one biologic disease-modifyin. Ann. Rheum. Dis. 77, 1276–1282. 34. Li, K.-J., Chang, C.-L., Hsin, C.-Y., and Tang, C.-H. (2021). Switching and Discontinuation Pattern of Biologic Disease-Modifying Antirheumatic Drugs and Tofacitinib for Patients With Rheumatoid Arthritis in Taiwan. Front. Pharmacol. 12. doi:10.3389/fphar.2021.628548. 35. Ljung, L., Rantapää-Dahlqvist, S., Jacobsson, L. T. H., and Askling, J. (2016). Response to biological treatment and subsequent risk of coronary events in rheumatoid arthritis. Ann. Rheum. Dis. 75, 2087–2094. 36. Màrquez Pete, N., Perez Ramirez, C., Maldonado Montoro, M. D. M., Espinosa Rodriguez, A., and Jimenez Morales, A. (2021b). Persistence of abatacept treatment in rheumatoid arthritis patients. Eur. J. Hosp. Pharm. 28, A141. 37. Martinez-MúGica, C., and Manso, G. (2020). Prescribing patterns and clinical outcomes of biological disease-modifying anti-rheumatic drugs for rheumatoid arthritis in Spain. Eur. Rev. Med. Pharmacol. Sci. 24, 8177–8184. doi:10.26355/EURREV_202008_22505. 38. Mothojakan, N. B., Gore, J., and Nisar, M. K. (2020). Does biologic survival depend on co-prescribed methotrexate dose in established rheumatoid arthritis? A real-world study. Eur. J. Rheumatol. 7, 21–25. doi:10.5152/eurjrheum.2019.19048. 39. Movahedi, M., Cesta, A., Li, X., Keystone, E., and Bombardier, C. (2020a). Time to discontinuation of tofacitinib and TNF inhibitors in rheumatoid arthritis patients with and without methotrexate: Data from a rheumatoid arthritis cohort. Ann. Rheum. Dis. 79, 131–132. 40. Movahedi, M., Cesta, A., Li, X., Keystone, E., and Bombardier, C. (2021). Discontinuation rate of Tofacitinib is similar when compared to TNF inhibitors in rheumatoid arthritis patients: Data from a rheumatoid arthritis cohort. Pharmacoepidemiol. Drug Saf. 30, 27–28. 41. Movahedi, M., Cesta, A., Li, X., Keystone, E. C., and Bombardier, C. (2020b). Discontinuation Rate of Tofacitinib Is Similar When Compared to TNF Inhibitors in Rheumatoid Arthritis Patients: Real World Results from a Rheumatoid Arthritis Cohort. Arthritis Rheumatol. 72, 1642–1643. 42. Movahedi, M., Hepworth, E., Mirza, R., Cesta, A., Larche, M., and Bombardier, C. (2020c). Discontinuation of biologic therapy due to lack/loss of response and adverse events is similar between TNFi and non-TNFi class: Results from a real-world rheumatoid arthritis cohort. Semin. Arthritis Rheum. 50, 915–922. doi:10.1016/j.semarthrit.2020.06.020. 43. Naffaa, M. E., Hassan, F., Golan-Cohen, A., Merzon, E., Green, I., Saab, A., et al. (2021). Factors associated with drug survival on first biologic therapy in patients with rheumatoid arthritis: a population-based cohort study. Rheumatol. Int. 41, 1905–1913. doi:10.1007/s00296-021-04989-y. 44. Neovius, M., Arkema, E. V, Olsson, H., Eriksson, J. K., Kristensen, L. E., Simard, J. F., et al. (2015). Drug survival on TNF inhibitors in patients with rheumatoid arthritis comparison of adalimumab, etanercept and infliximab. Ann. Rheum. Dis. 74, 354–360. 45. Nicholls, D., Barrett, R., Button, P., Truman, M., Bird, P., Roberts, L., et al. (2018). Effectiveness of biologics in Australian patients with rheumatoid arthritis: a large observational study: REAL. Intern. Med. J. 48, 1185–1192. doi:10.1111/imj.14028. 46. Ochi, S., Saito, K., Mizoguchi, F., Kato, S., and Tanaka, Y. (2020). Insensitivity versus poor response to tumour necrosis factor inhibitors in rheumatoid arthritis: a retrospective cohort study. Arthritis Res. Ther. 22, 41. doi:10.1186/s13075-020-2122-5. 47. Ochiai, M., Tanaka, E., Sato, E., Inoue, E., Abe, M., Saka, K., et al. (2021). Successful discontinuation of biological disease-modifying antirheumatic drugs in patients with rheumatoid arthritis in real-world settings. Mod. Rheumatol. 31, 790–795. 48. Østergaard, M., Unkerskov, J., Linde, L., Krogh, N. S., Ravn, T., Ringsdal, V. S., et al. (2007). Low remission rates but long drug survival in rheumatoid arthritis patients treated with infliximab or etanercept: results from the nationwide Danish DANBIO database. Scand. J. Rheumatol. 36, 151–154. doi:10.1080/03009740601089267. 49. Pappas, D. A., Blachley, T., Best, J. H., Zlotnick, S., Reiss, W. G., Emeanuru, K., et al. (2021a). Durability of Response to Tocilizumab Therapy in Rheumatoid Arthritis: Data from the US-Based Corrona Rheumatoid Arthritis Registry. Rheumatol. Ther. 8, 467–481. doi:10.1007/s40744-021-00285-0. 50. Pappas, D. A., Blachley, T., Zlotnick, S., Best, J. H., Emeanuru, K., and Kremer, J. M. (2019). Comparative effectiveness of tocilizumab in combination with methotrexate versus tumor necrosis factor inhibitors (TNFis) in combination with methotrexate in patients with rheumatoid arthritis with prior exposure to TNFis. Ann. Rheum. Dis. 71, 993. 51. Pappas, D. A., John, G. S., Etzel, C. J., Fiore, S., Blachley, T., Kimura, T., et al. (2020). Comparative effectiveness of first-line tumour necrosis factor inhibitor versus non-tumour necrosis factor inhibitor biologics and targeted synthetic agents in patients with rheumatoid arthritis: results from a large US registry study. Ann. Rheum. Dis. 52. Pappas, D. A., Litman, H. J., Lesperance, T., Kricorian, G., Karis, E., Rebello, S., et al. (2021b). Persistence on biologic DMARD monotherapy after achieving rheumatoid arthritis disease control on combination therapy: retrospective analysis of corrona registry data. Rheumatol. Int. 41, 381–390. doi:10.1007/s00296-020-04667-5. 53. Pappas, D. A., St John, G., Etzel, C. J., Fiore, S., Blachley, T., Kimura, T., et al. (2021c). Comparative effectiveness of first-line tumour necrosis factor inhibitor versus non-tumour necrosis factor inhibitor biologics and targeted synthetic agents in patients with rheumatoid arthritis: results from a large US registry study. Ann. Rheum. Dis. 80, 96–102. doi:10.1136/annrheumdis-2020-217209. 54. Paul, D., Han, X., Yermilov, I., Gibbs, S., and Broder, M. (2019). Evaluation of real-world early-line abatacept versus tumor necrosis factor inhibitors persistence in rheumatoid arthritis patients with Anti-Citrullinated Protein Antibody or Rheumatoid Factor Positivity. Arthritis Rheumatol. 71, 386–389. 55. Paul, S. K., Montvida, O., Best, J. H., Gale, S., Pethö-Schramm, A., and Sarsour, K. (2021). Association of biological antirheumatic therapy with risk for type 2 diabetes: a retrospective cohort study in incident rheumatoid arthritis. BMJ Open 11, e042246. doi:10.1136/bmjopen-2020-042246. 56. Sato, S., Matsumoto, H., Temmoku, J., Fujita, Y., Matsuoka, N., Yashiro-Furuya, M., et al. (2021). Sustained Long-Term Retention Rates of Abatacept in Combination with Conventional Synthetic Disease-Modifying Antirheumatic Drugs in Elderly Patients with Rheumatoid Arthritis. Medicina (B. Aires). 57, 914. doi:10.3390/medicina57090914. 57. Sebastiani, M., Anelli, M. G., Atzeni, F., Bazzani, C., Farina, I., Fedele, A. L., et al. (2014). Efficacy and safety of rituximab with and without methotrexate in the treatment of rheumatoid arthritis patients: Results from the GISEA register. Jt. Bone Spine 81, 508–512. doi:10.1016/j.jbspin.2014.06.011. 58. Sfikakis, P. P., Bournia, V. K., Sidiropoulos, P., Boumpas, D. T., Drosos, A. A., Kitas, G. D., et al. (2017). Biologic treatment for rheumatic disease: Real-world big data analysis from the Greek country-wide prescription database. Clin. Exp. Rheumatol. 35, 579–585. 59. Shidara, K., Hoshi, D., Inoue, E., Yamada, T., Nakajima, A., Taniguchi, A., et al. (2010). Incidence of and risk factors for interstitial pneumonia in patients with rheumatoid arthritis in a large Japanese observational cohort, IORRA. Mod. Rheumatol. 20, 280–286. doi:10.1007/s10165-010-0280-z. 60. Shivacheva, T., Georgiev, T., Hristova, S., Dimitrov, S., Bogdanova-Petrova, S., and Gerganov, G. (2021). Sustainable low disease activity in patients with rheumatoid arthritis: Real-world experience with tocilizumab. Ann. Rheum. Dis. 80, 1138. 61. Silvagni, E., Bortoluzzi, A., Carrara, G., Zanetti, A., Govoni, M., and Scirè, C. A. (2018). Comparative effectiveness of first-line biological monotherapy use in rheumatoid arthritis: a retrospective analysis of the RECord-linkage On Rheumatic Diseases study on health care administrative databases. BMJ Open 8, e021447. doi:10.1136/bmjopen-2017-021447. 62. Soliman, M. M., Ashcroft, D. M., Watson, K. D., Lunt, M., Symmons, D. P. M., and Hyrich, K. L. (2011). Impact of concomitant use of DMARDs on the persistence with anti-TNF therapies in patients with rheumatoid arthritis: results from the British Society for Rheumatology Biologics Register. Ann. Rheum. Dis. 70, 583–589. doi:10.1136/ard.2010.139774. 63. van Mulligen, E., Ahmed, S., Weel, A. E. A. M., Hazes, J. M. W., van der Helm- van Mil, A. H. M., and de Jong, P. H. P. (2021). Factors that influence biological survival in rheumatoid arthritis: results of a real-world academic cohort from the Netherlands. Clin. Rheumatol. 40, 2177–2183. doi:10.1007/s10067-020-05567-6. 64. Verhoeven, M. M. A., Tekstra, J., Welsing, P. M. J., Pethö-Schramm, A., Borm, M. E. A., Bruyn, G. A. W., et al. (2020). Effectiveness and safety over 3 years after the 2-year U-Act-Early trial of the strategies initiating tocilizumab and/or methotrexate. Rheumatology 59, 2325–2333. doi:10.1093/rheumatology/kez602. 65. Xie, F., Chen, L., Yun, H., Levitan, E. B., and Curtis, J. R. (2021). Benefits of Methotrexate Use on Cardiovascular Disease Risk Among Rheumatoid Arthritis Patients Initiating Biologic Disease-modifying Antirheumatic Drugs. J. Rheumatol. 48, 804–812. doi:10.3899/jrheum.191326. 66. Youssef, P., Marcal, B., Button, P., Truman, M., Bird, P., Griffiths, H., et al. (2020). Reasons for Biologic and Targeted Synthetic Disease-modifying Antirheumatic Drug Cessation and Persistence of Second-line Treatment in a Rheumatoid Arthritis Dataset. J. Rheumatol. 47, 1174–1181. doi:10.3899/jrheum.190535. 67. Yun, H., Xie, F., Delzell, E., Chen, L., Yang, S., Saag, K. G., et al. (2015). The comparative effectiveness of biologics among older adults and disabled rheumatoid arthritis patients in the Medicare population. Br. J. Clin. Pharmacol. 80, 1447–1457. doi:10.1111/bcp.12709. |
| **Wrong population** | 1. Alkhayyat, M., Abou Saleh, M., Grewal, M. K., Abureesh, M., Mansoor, E., Simons-Linares, C. R., et al. (2021). Pancreatic manifestations in rheumatoid arthritis: a national population-based study. Rheumatology 60, 2366–2374. doi:10.1093/rheumatology/keaa616. 2. Bhushan, V., Lester, S., Briggs, L., Hijjawi, R., Shanahan, E. M., Pontifex, E., et al. (2021). Real-Life Retention Rates and Reasons for Switching of Biological DMARDs in Rheumatoid Arthritis, Psoriatic Arthritis, and Ankylosing Spondylitis. Front. Med. 8. doi:10.3389/fmed.2021.708168. 3. Cronin, O., McKnight, O., Keir, L., Ralston, S. H., Hirani, N., and Harris, H. (2021). A retrospective comparison of respiratory events with JAK inhibitors or rituximab for rheumatoid arthritis in patients with pulmonary disease. Rheumatol. Int. 41, 921–928. doi:10.1007/s00296-021-04835-1. 4. Hsieh, M.-J., Lee, C.-H., Tsai, M.-L., Kao, C.-F., Lan, W.-C., Huang, Y.-T., et al. (2020). Biologic Agents Reduce Cardiovascular Events in Rheumatoid Arthritis Not Responsive to Tumour Necrosis Factor Inhibitors: A National Cohort Study. Can. J. Cardiol. 36, 1739–1746. doi:10.1016/j.cjca.2020.01.003. 5. Mena-Vázquez, N., Rojas-Gimenez, M., Romero-Barco, C. M., Manrique-Arija, S., Francisco, E., Aguilar-Hurtado, M. C., et al. (2021). Predictors of Progression and Mortality in Patients with Prevalent Rheumatoid Arthritis and Interstitial Lung Disease: A Prospective Cohort Study. J. Clin. Med. 10, 874. doi:10.3390/jcm10040874. 6. Plein, S., Erhayiem, B., Fent, G., Horton, S., Dumitru, R. B., Andrews, J., et al. (2020). Cardiovascular effects of biological versus conventional synthetic disease-modifying antirheumatic drug therapy in treatment-naïve, early rheumatoid arthritis. Ann. Rheum. Dis. 79, 1414–1422. doi:10.1136/annrheumdis-2020-217653. 7. Sugihara, T., Ishizaki, T., Onoguchi, W., Baba, H., Matsumoto, T., Iga, S., et al. (2021). Effectiveness and safety of treat-to-target strategy in elderly-onset rheumatoid arthritis: a 3-year prospective observational study. Rheumatology 60, 4252–4261. doi:10.1093/rheumatology/keaa922. 8. Watanabe, S., Gono, T., Fukue, R., Kobayashi, S., Shirai, Y., Takeno, M., et al. (2019). Treatment with biologic DMARDs does not increase risk of severe pulmonary events in patients with rheumatoid arthritis and preexisting lung disease. Arthritis Rheumatol. 71, 4133–4134. |
| **Insufficient data** | 1. Barbieri, M. A., Cicala, G., Cutroneo, P. M., Gerratana, E., Palleria, C., De Sarro, C., et al. (2020). Safety Profile of Biologics Used in Rheumatology: An Italian Prospective Pharmacovigilance Study. J. Clin. Med. 9, 1227. doi:10.3390/jcm9041227. 2. Calip, G. S., Patel, P. R., Adimadhyam, S., Xing, S., Wu, Z., Sweiss, K., et al. (2018). Tumor necrosis factor-alpha inhibitors and risk of non-Hodgkin lymphoma in a cohort of adults with rheumatologic conditions. Int. J. Cancer 143, 1062–1071. doi:10.1002/ijc.31407. 3. Celkys, K., Ly, J., and Soden, M. (2020). Serious infection rates with biological disease modifying anti-rheumatic agents (bDMARDs) and predisposing factors: A 5-year retrospective review. Ann. Rheum. Dis. 79, 1249–1250. 4. Delcoigne, B., Ljung, L., Provan, S. A., Glintborg, B., Lederballe Gron, K., Hetland, M. L., et al. (2021). Short-and longer-term risks for acute coronary syndrome in patients with rheumatoid arthritis starting treatment with disease-modifying anti-rheumatic drugs. a collaborative observational head-to-head study across five nordic rheumatology registers. Ann. Rheum. Dis. 80, 63–64. 5. Koike, T., Harigai, M., Ishiguro, N., Inokuma, S., Takei, S., Takeuchi, T., et al. (2012). Safety and effectiveness of adalimumab in Japanese rheumatoid arthritis patients: postmarketing surveillance report of the first 3,000 patients. Mod. Rheumatol. 22, 498–508. doi:10.1007/s10165-011-0541-5. 6. Krüger, K., Burmester, G. R., Wassenberg, S., Bohl-Bühler, M., and Thomas, M. H. (2018). Effectiveness and safety of golimumab in patients with rheumatoid arthritis, psoriatic arthritis and ankylosing spondylitis under real-life clinical conditions: non-interventional GO-NICE study in Germany. BMJ Open 8, e021082–e021082. 7. Màrquez Pete, N., Maldonado Montoro, M. D. M., Perez Ramirez, C., Espinosa Rodriguez, A., and Jimenez Morales, A. (2021a). Effectiveness and safety of abatacept therapy in patients with rheumatoid arthritis after previous failure with tnfi treatment. Eur. J. Hosp. Pharm. 28, A141–A142. 8. Moreland, L. W., Weinblatt, M. E., Keystone, E. C., Kremer, J. M., Martin, R. W., Schiff, M. H., et al. (2006). Etanercept treatment in adults with established rheumatoid arthritis: 7 years of clinical experience. J. Rheumatol. 33, 854–861. 9. Ogdie, A., Yu, Y., Haynes, K., Love, T. J., Maliha, S., Jiang, Y., et al. (2015). Risk of major cardiovascular events in patients with psoriatic arthritis, psoriasis and rheumatoid arthritis: a population-based cohort study. Ann. Rheum. Dis. 74, 326–332. 10. Osorio, P., Villarreal, L., Rivero, W., Ibata, L., Martinez, S., Rojas-Villarraga, A., et al. (2021). Certolizumab in monotherapy as effective than in combination in rheumatoid arthritis patients. Ann. Rheum. Dis. 80, 1146. 11. Rotar, Z., Svetina, P., Tomsic, M., Hočevar, A., and Prapotnik, S. (2020). Tuberculosis among patients treated with TNF inhibitors for rheumatoid arthritis, ankylosing spondylitis and psoriatic arthritis in Slovenia: a cohort study. BMJ Open 10, e034356–e034356. 12. Seror, R., Lafourcade, A., De Rycke, Y., Fautrel, B., Mariette, X., and Tubach, F. (2019). Risk of malignancies associated with biologics in rheumatoid arthritis: Analysis of a national claim database. Arthritis Rheumatol. 71, 1466–1467. 13. Winthrop, K. L., Saag, K., Cascino, M. D., Pei, J., John, A., Jahreis, A., et al. (2019). Long-Term Safety of Rituximab in Patients With Rheumatoid Arthritis: Results of a Five-Year Observational Study. Arthritis Care Res. (Hoboken). 71, 993–1003. doi: 10.1002/acr.23781. |

**Table S3.** Additional characteristics of the included studies.

| **Study** | **Year** | **Study design** | **Secondary data sources** | **Treatment** | **Control** | **Age** | **Adjustment** | **Comorbidities** | **RA assessment** | **Outcome assessment** |
| --- | --- | --- | --- | --- | --- | --- | --- | --- | --- | --- |
| Arkema | 2014 | Population-based cohort 2002-2011 | Swedish Rheumatology Quality Register (SRQ) | Biological-exposed RA (10,800) | General population (175,972)  Biological-naïve RA (37,982) | Biological-naïve RA  63.1±14.1;  Biological-exposed RA  56.4±13.3 | Sex, age, education, country of birth and history of any visit with an ICD code for diabetes, cancer, chronic obstructive pulmonary disease | Diabetes, cancer, and chronic obstructive pulmonary disease | ICD code | Tuberculosis Register |
| Chen | 2020 | Population-based cohort 2006-2015 | Truven MarketScan database | Abatacept (11,248) | TNFi  (11,248). | Abatacept  (55.3 ± 12.8) TNFi  (55.5 ± 12.7) | Age, sex, calendar year of index date, region of residence, comorbidities, and hospitalization for infection by ICD-9 codes, and calculated combined comorbidity index at baseline. | Hypertension, diabetes mellitus, obesity, smoking, alcohol  use, depression, cardiovascular disease, chronic renal disease,  chronic liver disease, pulmonary disease, viral hepatitis, inflammatory  bowel disease, and hospitalization for infection | ICD-9 code 714.xx | ICD-9 codes |
| Chen | 2021 | Population-based case-control 1995-2014 | National Health Insurance Research Database (NHIRD) | bDMARDs - MACE (113) Etanecept (49) Adalimumab (45) Golimumab (1) Tocilizumab (3) Abatacept (1) Rituximab (14) | bDMARDs patients (917) Etanecept (377) Adalimumab (274) Golimumab (68) Tocilizumab (42) Abatacept (49) Rituximab (107) | 69.19 ± 11.62 | Age, gender, disease duration, and comorbidities by ICD-9 codes, Major adverse cardiovascular events (MACE): composite of myocardial infarction (MI), coronary revascularization, ischemic stroke, and cardiovascular death, | Heart failure, hypertension, diabetes mellitus, vascular disease, hyperlipidemia, ischemic heart disease, valvular heart disease, chronic  obstructive pulmonary disease, renal disease, hyperthyroidism, depression, and anxiety | ICD-9 code 714.0 | ICD-9 codes 410 (except for 410.x2 for inpatients  with a hospitalization for at least 3 days, unless  mortality occurred), 00.66, 36.03, 36.06, 36.07,  36.1, 36.2, and 433-436 (except 433.x0 and 434.x0, for ischemic stroke) |
| Curtis | 2016 | Retrospective cohort | Medicare (2006–2013) and Marketscan (2010–2014) | Abatacept (11,434) Rituximab (4,785) TNFi (38,871) Tocilizumab (6,266) | Tofacitinib (1,746) | 57.1-62.0 | Age, sex, baseline glucocorticoid use, methotrexate, number of biologics used, hospitalization, hospitalized infection, outpatient infection, and zoster vaccination | Diabetes mellitus, Chronic obstructive pulmonary disease, Heart failure, Renal disease, Any fracture, and Hospitalized infections during baseline | ICD-9 codes 714.0, 714.2, 714.81 | ICD9 code 053.xx |
| Desai | 2017 | Population-based cohort 2001-2012 | WellPoint and United HealthCare | TNF-α inhibitor (4,822) | Non-biologic DMARD  (2,400) | TNF-α inhibitor  (46) Non-biologic DMARD  (48) | Age, gender, diabetes, hyperlipidemia, lipid-lowering agent use, anti-diabetic medication use, obesity, smoking, combined comorbidity score, use of non-steroidal anti-inflammatory drugs, injectable steroids, cumulative dose of oral steroids, methotrexate use, leflunomide or cyclosporine use, other non-TNF biologic use, hospitalizations, emergency room visits, office visits, and number of distinct drugs | Diabetes, hyperlipidemia, and obesity | ICD-9 code 714.xx | ICD-9 code 401.xx and a new prescription for an antihypertensive agent |
| Dreyer | 2017 | Population-based cohort 2000-2011 | Danish DANBIO Registry; Danish Cancer Registry2000–2011 | bDMARDs  (502) | Non bDMARDs (1,176) | bDMARDs: 65 (32-86) Non bDMARDs: 70 (28-96) | Age, sex, calendar time (2000–2004, 2005–2009, 2010–2011), and cancer site | - | - | ICD-7 and 10 codes |
| de Germay | 2020 | Case-control  2007-2017 | Global Health Organization Global Individual Case Safety Reports (ICSRs) | Abatacept  (931) | Other bDMARDs (15,261) | Abatacept: 60.2 Other bDMARDs: 57.6 | Age, sex and medications (conventional DMARD and others that could induce a cancer) | - | - | All patients with a report  related to cancer and classified as “malignant or unspecified tumours” by standardized MedDRA queries (SMQs) |
| Grøn | 2019 | Prospective cohort 2010-2015 | Danish nationwide quality registry (DANBIO) Swedish biologics register (ARTIS) | Abatacept (2,725) | Rituximab  (3,363) Tocilizumab  (2,899) | DANBIO Abatacept: 59 Rituximab: 61 Tocilizumab: 59  ARTIS Abatacept: 61 Rituximab: 64 Tocilizumab: 59 | Age and gender | Malignancy, Chronic obstructive or interstitial pulmonar disease, Diabetes, Myocardial infarction, and Chronic kidney disease | - | ICD-10 code A00-B99, D73.3, E06.0, E32.1, G00-G02, G04.2, G05-G07, H00.0, H44.0, H60.0-H60.3, H66-H67, H70, I30.1, I40.0, J00-J22, J32, J34.0, J36, J38.3, J39.0-J39.1, J44.0, J85, J86, K04.4, K04.6, K04.7, K10.2, K11.3, K12.2, K14.0, K57.0, K57.2, K57.4, K57.8, K61, K63.0, K65.0, K65.1, K65.2, K65.9, L00-L08, L30.3, M00-M01, M46.2-M46.5, M60.0, M65.0, M71.0, M71.1, M72.6, M86, N10, N11, N12, N13.6, N15.1, N15.9, N30.0 N30.8, N34.0, N39.0, N41.2, N43.1, N45.2, N45.3, N45.4, N48.2, N61, N70, N73, N75.1 O23, O26.4, O41.1, O75.3, O85, O86, O88.3, O91, and O98 |
| Grøn | 2020 | Prospective cohort 2007 - 2017 | Danish nationwide quality registry (DANBIO) | Tocilizumab  (2,899) | Abatacept  (2,725) Rituximab (3,363) | Abatacept: 59 Rituximab: 62 Tocilizumab: 59 | Age, gender, calendar year, RA disease duration (years), number of previous bDMARDs, glucocorticoid use, DAS28, disease duration, HAQ, smoking, previous malignancy, previous hospitalized infection, previous chronic obstructive or interstitial pulmonary disease, and previous reimbursement of an antibiotic prescription | Malignancy, Chronic obstructive or interstitial pulmonar disease, Diabetes, Myocardial infarction, and Chronic kidney disease | - | ICD-10 code A00-B99, D73.3, E06.0, E32.1, G00-G02, G04.2, G05-G07, H00.0, H44.0, H60.0-H60.3, H66-H67, H70, I30.1, I40.0, J00-J22, J32, J34.0, J36, J38.3, J39.0-J39.1, J44.0, J85, J86, K04.4, K04.6, K04.7, K10.2, K11.3, K12.2, K14.0, K57.0, K57.2, K57.4, K57.8, K61, K63.0, K65.0, K65.1, K65.2, K65.9, L00-L08, L30.3, M00-M01, M46.2-M46.5, M60.0, M65.0, M71.0, M71.1, M72.6, M86, N10, N11, N12, N13.6, N15.1, N15.9, N30.0 N30.8, N34.0, N39.0, N41.2, N43.1, N45.2, N45.3, N45.4, N48.2, N61, N70, N73, N75.1 O23, O26.4, O41.1, O75.3, O85, O86, O88.3, O91, and O98 |
| Harada | 2017 | Nested case-control 2005-2012 | Registry of Japanese Rheumatoid Arthritis Patients on Biologics for Longterm Safety (REAL) | bDMARDs (1,222) | MTX  (1,274) | 60.0 (51.0-68.0) | Age, sex, observation start year and comorbidity | Pulmonary disease, Liver disease, Renal disease, Diabetes mellitus, and Malignancies | - | Clinical diagnose by the site investigators and patients that required intravenous or oral  administration of antiviral medications |
| Hellgren | 2020 | Population-based cohort 2001-2016. | Swedish Rheumatology Quality Register (SRQ) | bDMARDs (16,392) | ccsDMARDss (55,253) | bDMARDs: 57 (18-92) ccsDMARDss: 62 (18-97) | Age, sex, educational level and comorbidities | Chronic obstructive pulmonary disease, Ischemic heart disease, and Diabetes mellitus | ICD-10 code M05, M060, M062, M063, M068, M069, and M123 | ICD-10 code C82, C83, C84, C85, C86, C88, C911, C913, C916 , C914, C97.7, C96.7, C96.2, C911, C913,C916, and C81 |
| Kim | 2017 | Retrospective cohort | Medicare (2010–2013), IMS PharMetrics Plus (2011–2014), and Truven MarketScan (2011–June 2015) | TNFi  (530,202) | Tocilizumab (59,917) | 51.0-72.0 | Age, sex, region, race/ethnicity, prior DMARD use, cardiovascular comorbidities, other chronic diseases, cardiovascular medications, other long-term medications, and markers of health care utilization intensity | Atrial fibrillation, Myocardial infarction, Acute/subacute coronary artery disease, Chronic coronary artery disease, Atherosclerosis, Heart failure, Stroke, transient ischemic attack, Peripheral vascular disease, Hypertension, Diabetes, Hyperlipidemia, and Chronic kidney disease | ICD-9 code 714.x | ICD-9 code 410.x (excluding 410.x2), 430, 431,  433.x1, 434.x1, and 436 |
| Kim | 2020 | Population-based cohort 2012-2018. | Korean College of Rheumatology Biologics & Targeted Therapy (KOBIO) registry | bDMARDs  (615) | ccsDMARDss (381) | 56.1 | - | Dyslipidemia and Diabetes mellitus | American College of Rheumatology revised classification criteria | Sistolic Blood Pressure greater than 140mm Hg and/or Diastolic Blood Pressure greater  than 90mm Hg;  Use of new antihypertensive  drugs such as b-blockers, calcium channel blockers, angiotensin converting enzyme inhibitors, or angiotensin II receptor blockers during the follow-up period |
| Listing | 2015 | Prospective cohort 2001-2011 | RABBIT Register | csDMARDs  (928) TNFα inhibitors  (4,649) Rituximab  (703) Other biologics  (568) | MTX  (2,060) | 55.8^ⴕ^ | Age, sex, smoking, diabetes, chronic lung disease, chronic renal disease, prior malignancy, osteoporosis, coronary heart disease, DAS28 score, treatment with glucocorticoids, methotrexate, other synthetic disease modifying antirheumatic drugs, rituximab, or other biologics | Diabetes, Coronary heart disease, Chronic lung disease, Chronic renal disease, Prior malignancy, and Osteoporosis | - | - |
| Low | 2017 | Population-based cohort 2001-2009 | The British Society for Rheumatology Biologics Register | TNFα inhibitors (11,200) | csDMARDs (3,058) | TNFα inhibitors: 55.6 csDMARDs: 59.5 | Age, gender, DAS28 score, disease duration, health assessment questionnaire score, use of four or more csDMARDss prior to study registration, time of recruitment, hypertension, diabetes, chronic lung disease, smoking, antiplatelet therapy, NSAID/COX-2 inhibitor use, glucocorticoid use, and statin use. | Hypertension, Diabetes, and Chronic lung disease | - | History of  MI or angina (physicians), information on hospitalisations  via questionnaires every 6 months (patients), all reports of MI, additional clinical data (discharge  summaries, ECG, cardiac enzymes, pathology reports), and causes of death were coded from ICD-10 |
| Meissner | 2017 | Prospective cohort 2001-2015 | RABBIT Register | TNFα inhibitors  (238) | Other bDMARD and csDMARDs  (251) | Cases: 63.4 Controls: 62.6 | Age, gender, hypertension, coronary heart disease, heart failure, diabetes, smoking, and enrolment episode | Hypertension, Coronary heart disease, and Diabetes mellitus | - | International Council for  Harmonisation (ICH) |
| Mercer | 2015 | Population-based cohort 2001-2011 | The British Society for Rheumatology Biologics Register | TNFα inhibitors (11,767) | csDMARDs (3,249) | TNFα inhibitors: 56.0 csDMARDs: 60.0 | Age, sex, smoking history, ethnicity, comorbidity, DAS28 score, HAQ score, RA duration, number of previous csDMARDss, exposure to glucocorticoids at baseline, prior exposure to azathioprine, prior exposure to cyclophosphamide, and registration date | Hypertension, ischaemic heart disease, stroke, asthma, bronchitis or emphysema, diabetes mellitus, depression, renal disease, and liver disease | - | Nurse completed  Questionnaires, 6-monthly patient health diaries, and ICD-10 |
| Mercer | 2017 | Population-based cohort 2001-2013 | The British Society for Rheumatology Biologics Register | TNFα inhibitors  (11,931) | csDMARDs  (3,367) | TNFα inhibitors: 56.0 csDMARDs: 60.0 | Age, sex, smoking status, ethnicity, prior cancer (excluding LPM or MPM), comorbidity, DAS28 score, HAQ score, RA duration, number of previous csDMARDss, current exposure to corticosteroids, and date of registration | Hypertension, ischaemic heart disease, stroke, lung disease, diabetes mellitus, depression, renal disease, and liver disease | - | Histology reports and ICD-10 codes |
| Ozen | 2021 | Prospective cohort 1998-2017. | The National Databank for Rheumatic Diseases (FORWARD) | TNFα inhibitors  (7,724)  non-TNFα inhibitors (2,574) | csDMARDs  (15,541) | 58.6 | Age, sex, disease duration, socioeconomic status (employment and education level, insurance, location of residency), ethnicity, smoking, hypertension, diabetes, comorbidity index, BMI, HAQ, NSAIDs, statins, prior count of csDMARDs and bDMARDs, prior CVD history and year of entry | Diabetes, Hypertension, and Pulmonary disease | - | ICD-9/10: 410, I21, I22, 433, 434, I63, 428, I50 |
| Patel | 2021 | Retrospective cohort  2009-2017 | Medicare | Abatacept  (6,303) | TNFi  (18,032) Other non-TNFi (6,104) | Abatacept: 73.6 TNFi: 72.8 Other non-TNFi: 73.1 | Demographic and clinical characteristics | Congestive heart failure, Hypertension, Chronic obstructive  pulmonary disease, Chronic liver disease, Neutropenia, Renal disease, and Diabetes | ICD-9 and 10 | Inpatient medical services diagnosis codes  in any position for all types of infections |
| Pawar | 2019 | Retrospective cohort | Medicare (2010-2015), IMS PharMetrics (2011-2015), and Truven MarketScan (2011-2015) | Tocilizumab  Medicare: (5,827)  IMS PharMetrics: (4,642) Truven ‘MarketScan’TnFi: (5,605) | Abatacept  Medicare: (10,716)  IMS ‘PharMetrics’: (10,333)  Truven ‘MarketScan’TnFi: (12,060) | Medicare: 72.0  IMS: 51.0  MarketScan: 53.0 | Index year, demographics, comorbid conditions, combined comorbidity index, claim-based index of RA severity index, use of DMARDs and other prescription drugs, vaccination, and history of any invasive procedures or surgery; Markers of healthcare utilisation intensity, including receipt of cancer screening tests and physician orders of outpatient laboratory tests for acute phase reactants. | Atrial fibrillation, Coronary artery disease, Heart failure, Stroke/transient ischaemic attack, peripheral vascular disease,  Hypertension, Diabetes, Hyperlipidaemia, chronic  kidney disease | ICD-9 and 10 | Principal discharge diagnosis |
| Pawar | 2020 | Retrospective cohort | Medicare (2012-2015) Optum Clinformatics (2012-2018) IBM MarketScan (2012-2017) | Tofacitinib (6,278) | bDMARDs (124,440) | Medicare: 72.0 Optum: 54.0 MarketScan: 52.0 | - | Coronary artery disease, Hypertension, Diabetes, Hyperlipidaemia, Heart failure, Renal dialysis or chronic  kidney disease, Chronic liver disease,  Asthma, and Chronic obstructive  pulmonary disease | ICD-9 and 10 | ICD-9 and 10 |
| Pettipher and Benitha | 2019 | Population-based cohort 2008-2017 | South African Biologics Registry (SABIO) | 1,587 | 152 | Biologic exposed: 52.0 Biologic naïve: 50.7 | Gender, disease category, race, region, comorbidities, age | - | - | Culture, Gen Probe PCR, smear acid fast  bacilli (AFB), histological or radiographic confirmation or clinical suspicion |
| Raaschou | 2014 | Population-based cohort 2001-2010 | Swedish biologics register (ARTIS) | TNFi-treated  (120) | Biologics-naive (120) | Biologics-naive 67 (11) TNFi-treated 66 (11) | Age at diagnosis, year of diagnosis, county of residency and stage at diagnosis of index cancer | Chronic obstructive pulmonary disease, Diabetes mellitus, and Ischaemic heart disease | - | ICD7 through 10 and ICD-O classifications |
| Rahman | 2020 | Prospective cohort 2002-2017 | The Biologic Treatment Registry Across Canada (BioTRAC) | Golimumab (687) | Infliximab (890) | 55.8–57.7 | - | - | - | - |
| Richter | 2016 | Prospective cohort 2001-2013 | RABBIT biologics register | TNFi (370) | Other bDMARDS (159)  ccsDMARDsS  (388) | 59-68.2 | Age, sex, comorbid conditions, physical function and glucocorticoid dose | Heart failure Chronic renal disease, Diabetes, and Chronic obstructive pulmonary disease | - | Serious infection according to International Council for  Harmonisation of Technical Requirements for Registration of  Pharmaceuticals for Human Use (ICH) |
| Rutherford | 2018 | Prospective cohort 2010-2016 | British Society for Rheumatology Biologics Register for Rheumatoid Arthritis | Etanercept  (8,630) Infliximab  (4,908) Adalimumab  (7,818) Certolizumab (1,446) | Rituximab  (5,101) Tocilizumab (2,174) | 56-60 | Age, sex, DAS28-ESR, HAQ, disease duration, smoking, seropositivity, polypharmacy, baseline steroid usage and poly-pharmacy as a surrogate for comorbidity | Asthma, Diabetes, and Chronic obstructive pulmonary disease | - | Patient questionnaires, physician questionnaires or  death certificates |
| Sakai | 2018 | Nested case-control 2005-2013 | Japanese health insurance database | TNFi (129) | Non-TNFi  (35) | 55 | Previous Herpes-Zoster infection, renal disease and diabetes | Chronic pulmonary  disease, Renal disease, and Diabetes mellitus | ICD-10 code M05, M060, M062, M063, M068, and M069 | ICD-10 code B02  Prescription of antiviral  drugs for Herpes Zoster in the same month during the observation period |
| Yun | 2015 | Retrospective cohort 2006-2010 | Centers for Medicare and Medicaid Services (CMS) | Adalimumab (1,423) Etanercept (1,831) Rituximab (133) | Abatacept (333) Infliximab (4,087) | 64-69 | Demographics, comorbidities, concurrent medications, and health service utilization | Diabetes, Chronic obstructive pulmonary disease, Heart failure, Angina, and  Renal disease | ICD-9 code 714.x | Hospital diagnosis codes for infections in any position |
| Yun | 2016 | Retrospective cohort 2006-2011 | Medicare administrative data | Adalimumab (4,845) Certolizumab  (1,866) Etanercept (3,814) Golimumab  (1,394) Infliximab  (3,944) Rituximab (4,718) Tocilizumab  (2,016) | Abatacept  (9,204) | 60.4-66.8 | Infection risk score according to factors: age, sex, comorbities, medications, health behaviors and health service utilization, and reasons for receiving Medicare | Diabetes mellitus,  Chronic obstructive pulmonary disease,  Heart failure,  Renal disease, and  Peptic ulcer disease | ICD-9 code 714.x | Inpatient physician discharge diagnosis codes  in any position for all types of infections |
| Zhang | 2016 | Retrospective cohort  2006-2012 | Medical and pharmacy claims from Medicare data | Adalimumab  (10,241) Certolizumab  (2,956) Etanercept (9,763) Golimumab  (1,774) Infliximab  (12,758) Rituximab (7,475) Tocilizumab  (3,332) | Abatacept (13,608) | 64 | Age, sex, race, original reason for Medicare enrolment, receipt of subsidised Medicare premium, CV risk factors, other comobid diseases, and use of CV medication | Diabetes, Hypertension, and Hyperlipidaemia | ≥2 diagnosis codes  for RA from a physician that were between 7 days and  12 months apart | Acute myocardial infarction: ICD-9 410 |

**Table S4.** Methodological quality assessment according to the Newcastle-Ottawa Scale.

| **Study** | **Selection** | **Comparability** | **Outcome** | **Total score** | **Methodological quality** |
| --- | --- | --- | --- | --- | --- |
| Arkema et al. (2014) | **** | ** | ** | 8 | High |
| Chen et al. (2020) | **** | ** | *** | 9 | High |
| Chen et al. (2021) | **** | ** | ** | 8 | High |
| Curtis et al. (2016) | **** | * | ** | 7 | High |
| Desai et al. (2017) | **** | ** | ** | 8 | High |
| Dreyer et al. (2017) | **** | * | *** | 8 | High |
| de Germay et al. (2020) | *** | * | *** | 7 | High |
| Gron et al. (2019) | **** | * | *** | 8 | High |
| Gron et al. (2020) | **** | ** | ** | 8 | High |
| Harada et al. (2017) | *** |  | *** | 6 | Moderate |
| Hellgren et al. (2020) | **** | ** | *** | 9 | High |
| Kim et al. (2017) | *** | ** | *** | 8 | High |
| Kim et al. (2020) | **** | * | ** | 7 | High |
| Listing et al. (2015) | **** | ** | ** | 8 | High |
| Low et al. (2017) | **** | ** | ** | 8 | High |
| Meissner et al. (2017) | **** | ** | *** | 9 | High |
| Mercer et al. (2015) | **** | ** | *** | 9 | High |
| Mercer et al. (2017) | **** | ** | *** | 9 | High |
| Ozen et al. (2021) | **** | ** | ** | 8 | High |
| Patel et al. (2021) | *** | ** | ** | 7 | High |
| Pawar et al. (2019) | **** | ** | *** | 9 | High |
| Pawar et al. (2020) | **** | ** | ** | 8 | High |
| Pettipher and Benitha (2019) | ** |  | ** | 4 | Moderate |
| Raaschou et al. (2014) | **** | * | ** | 7 | High |
| Rahman et al. (2020) | **** |  | * | 5 | Moderate |
| Richter et al. (2016) | **** | ** | ** | 8 | High |
| Rutherford et al. (2019) | **** | * | *** | 8 | High |
| Sakai et al. (2018) | *** | * | ** | 6 | Moderate |
| Yun et al. (2014) | **** | * | *** | 8 | High |
| Yun et al. (2016) | **** | * | *** | 8 | High |
| Zhang et al. (2016) | **** | ** | *** | 9 | High |

*: the study has met the criteria for a domain of the Newcastle-Ottawa Scale. Each star scores one point.


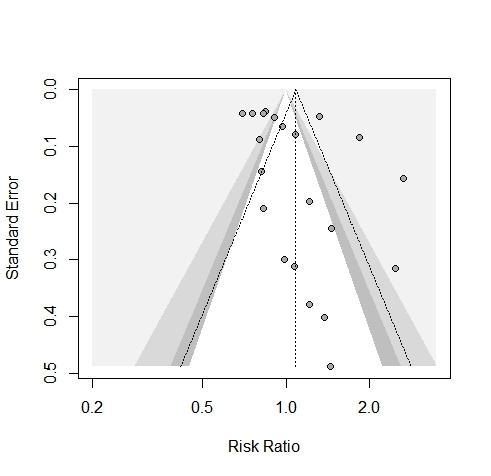


**Figure S1.** Funnel plot of studies that compared the safety of TNFi versus non-TNFi.


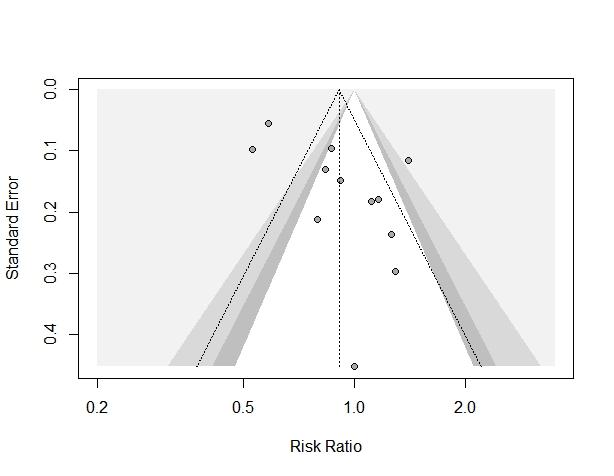


**Figure S2.** Funnel plot of studies that compared the safety of TNFi versus csDMARDs.


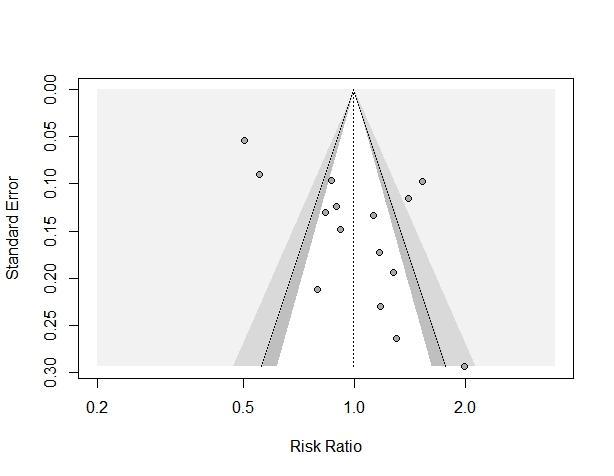


**Figure S3.** Funnel plot of studies that compared the safety of bDMARDs versus csDMARDs.


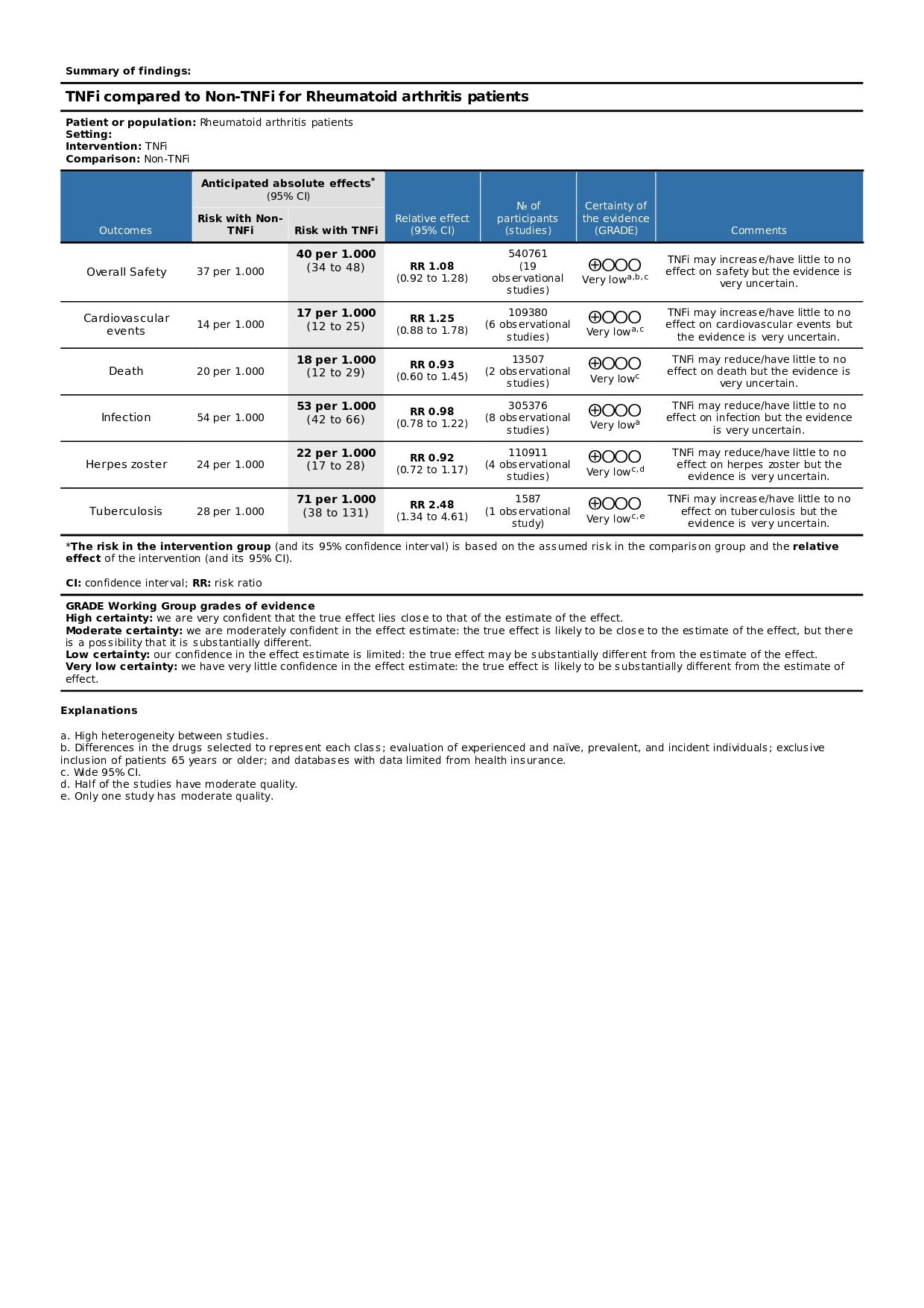


**Figure S4.** GRADE assessment of the methodological quality of studies that evaluated TNFi versus non-TNFi.


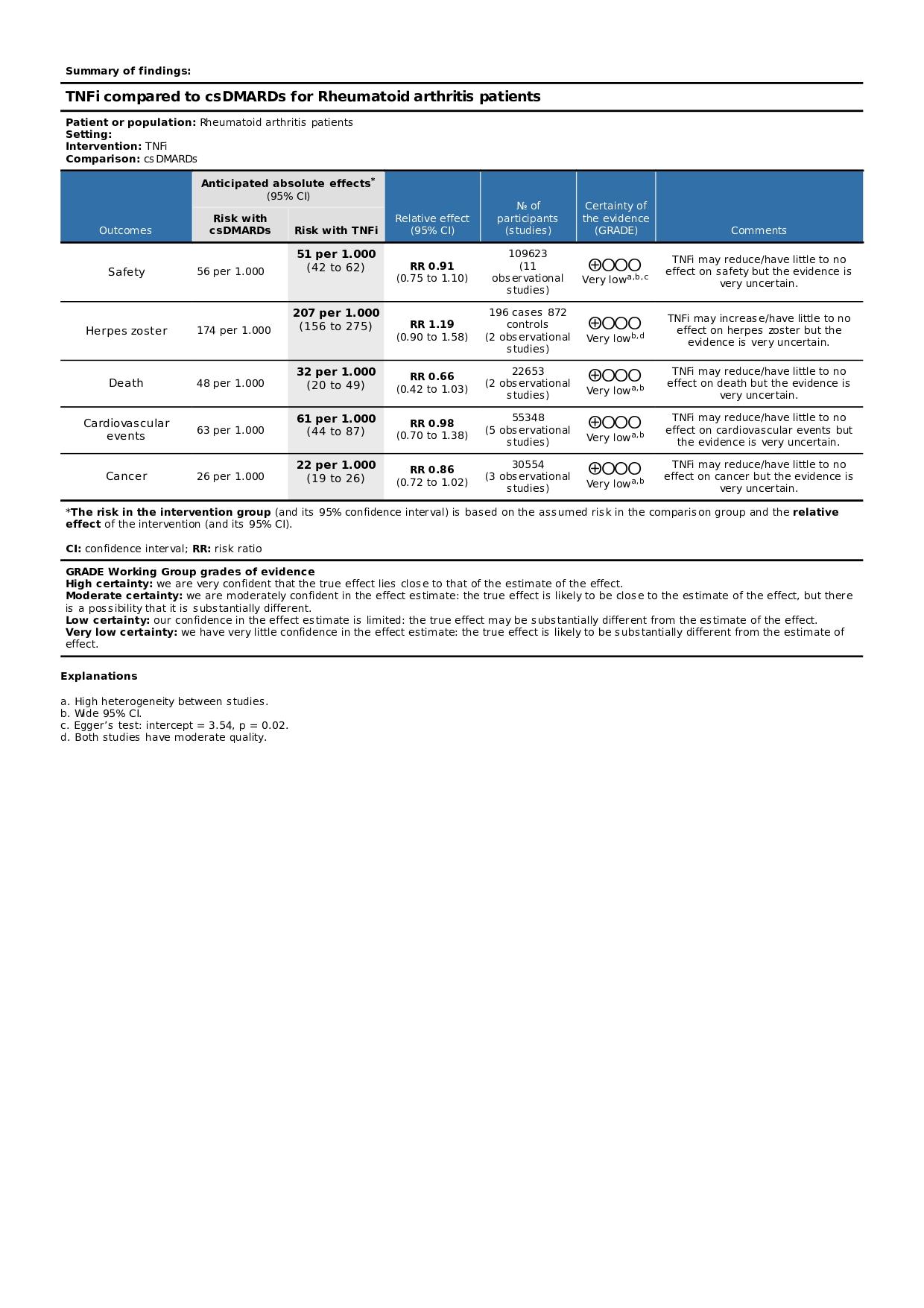


**Figure S5.** GRADE assessment of the methodological quality of studies that evaluated TNFi versus csDMARDs.


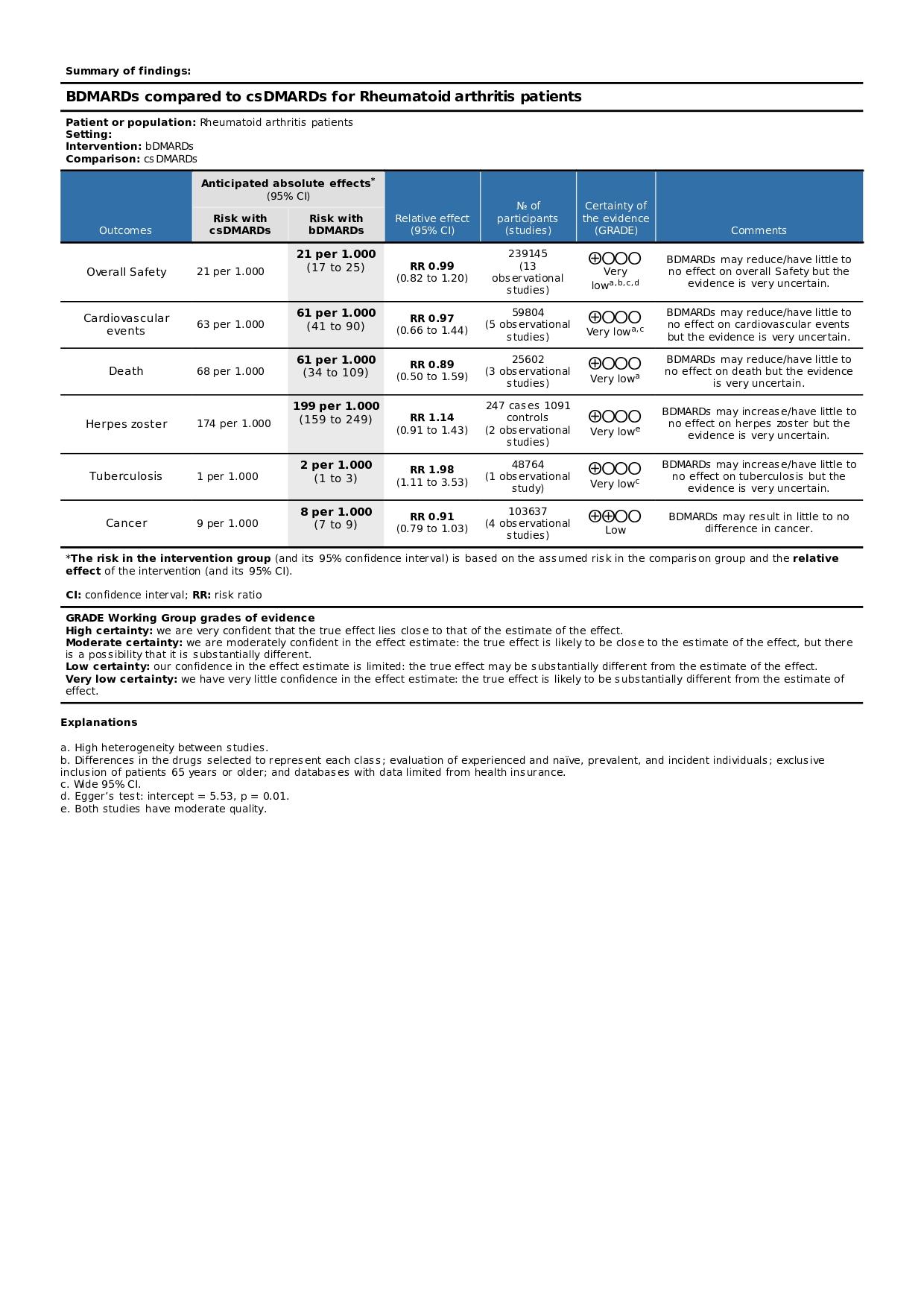


**Figure S6.** GRADE assessment of the methodological quality of studies that evaluated bDMARDs versus csDMARDs.


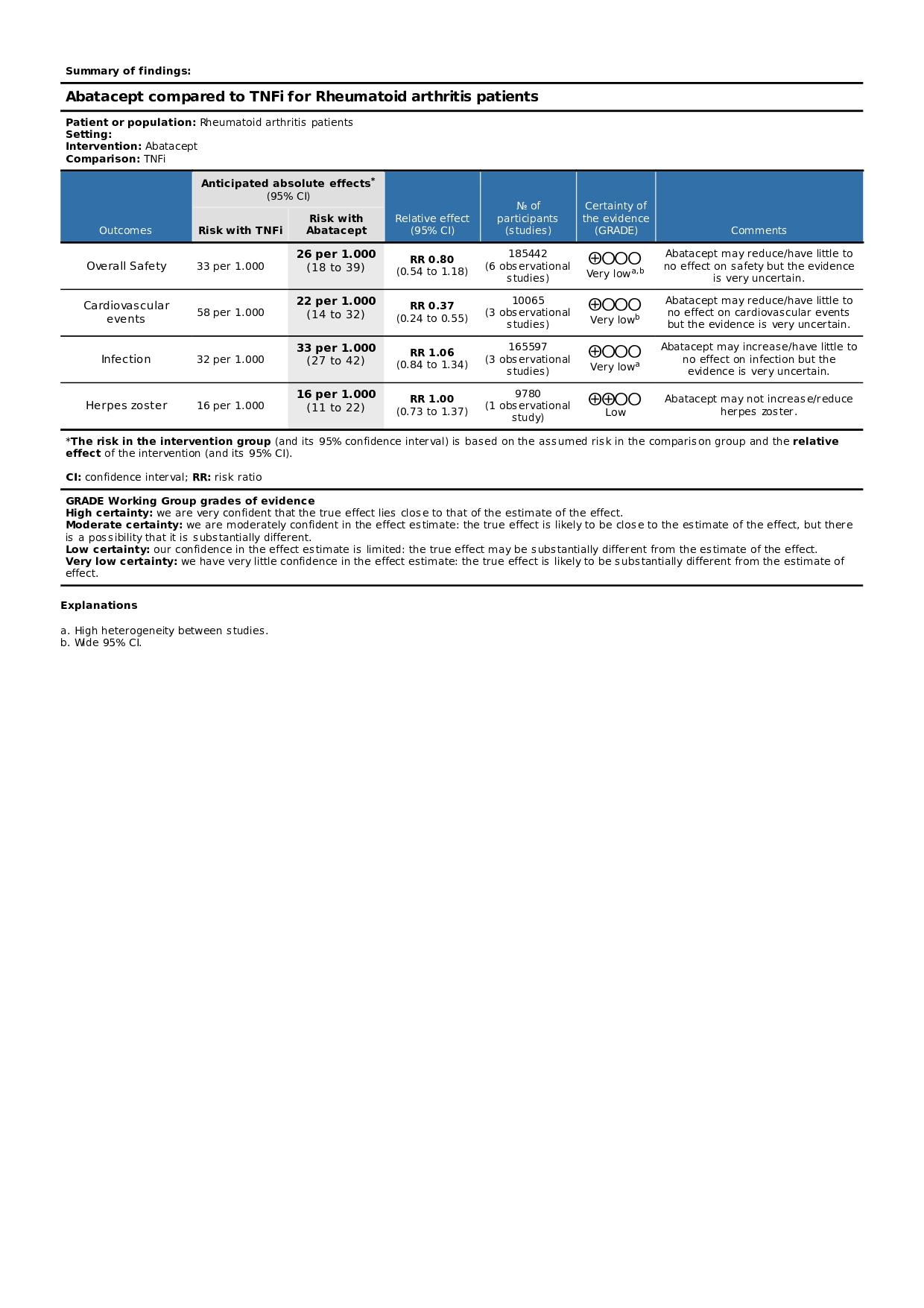


**Figure S7.** GRADE assessment of the methodological quality of studies that evaluated Abatacept versus TNFi.


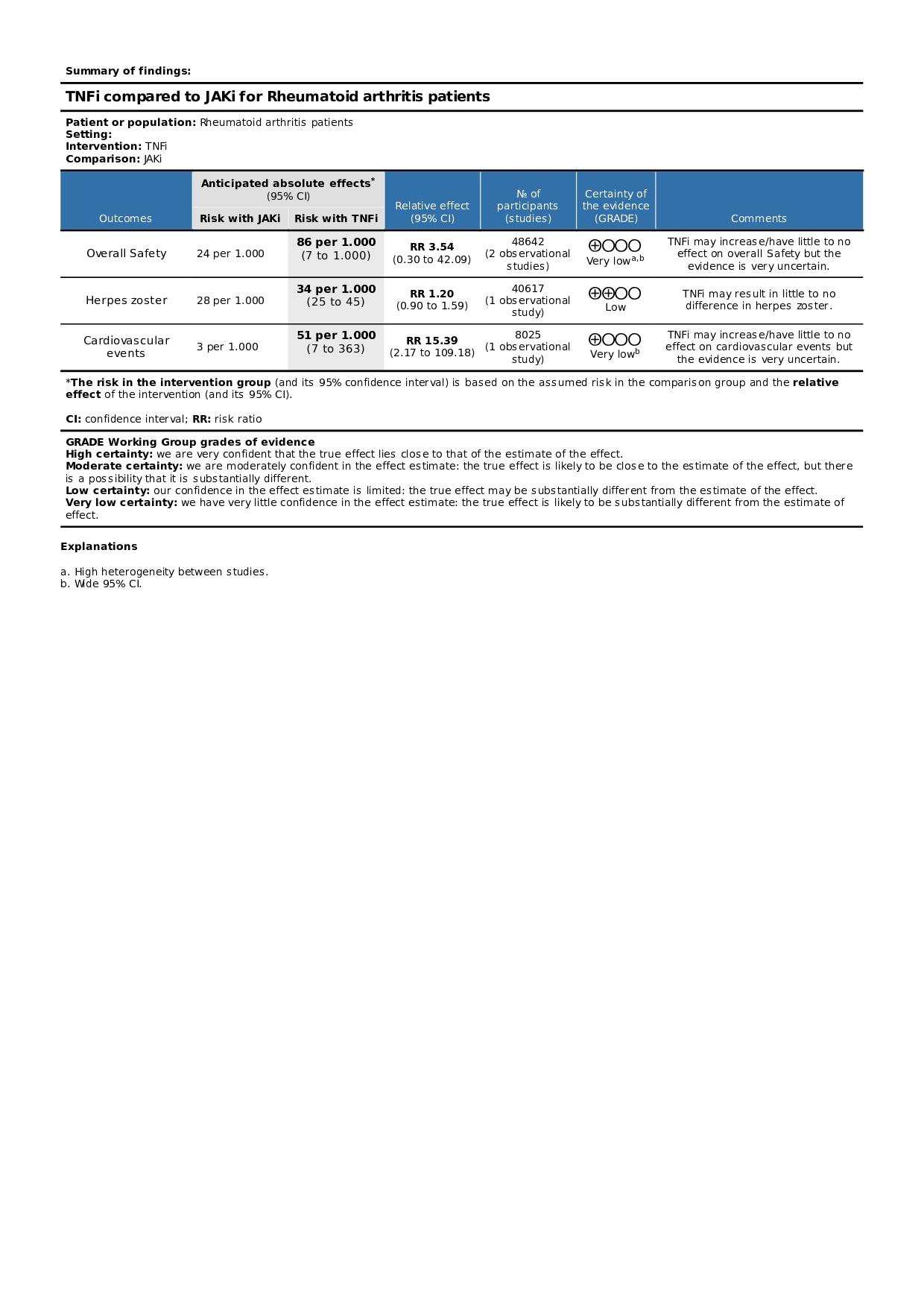


**Figure S8.** GRADE assessment of the methodological quality of studies that evaluated TNFi versus JAKi.
